# Association mapping reveals novel genes and genomic regions controlling grain size architecture in mini core accessions of Indian National Genebank wheat germplasm collection

**DOI:** 10.3389/fpls.2023.1148658

**Published:** 2023-06-28

**Authors:** Jyoti Kumari, Deepika Lakhwani, Preeti Jakhar, Shivani Sharma, Shailesh Tiwari, Shikha Mittal, Himanshu Avashthi, Neelam Shekhawat, Kartar Singh, Kaushlesh Kumar Mishra, Rakesh Singh, Mahesh C. Yadav, Gyanendra Pratap Singh, Amit Kumar Singh

**Affiliations:** ^1^ ICAR-National Bureau of Plant Genetic Resources, New Delhi, India; ^2^ Jaypee University of Information Technology, Solan, India; ^3^ ICAR-National Bureau of Plant Genetic Resources, Regional Station, Jodhpur, Jodhpur, India; ^4^ Zonal Agricultural Research Station, Powarkheda, India

**Keywords:** wheat, QTN, genome wide association studies, thousand grain weight, mrMLM

## Abstract

Wheat (*Triticum aestivum L*.) is a staple food crop for the global human population, and thus wheat breeders are consistently working to enhance its yield worldwide. In this study, we utilized a sub-set of Indian wheat mini core germplasm to underpin the genetic architecture for seed shape-associated traits. The wheat mini core subset (125 accessions) was genotyped using 35K SNP array and evaluated for grain shape traits such as grain length (GL), grain width (GW), grain length, width ratio (GLWR), and thousand grain weight (TGW) across the seven different environments (E_1_, E_2_, E_3_, E_4_, E_5_, E_5_, E_6_, and E_7_). Marker-trait associations were determined using a multi-locus random-SNP-effect Mixed Linear Model (mrMLM) program. A total of 160 non-redundant quantitative trait nucleotides (QTNs) were identified for four grain shape traits using two or more GWAS models. Among these 160 QTNs, 27, 36, 38, and 35 QTNs were associated for GL, GW, GLWR, and TGW respectively while 24 QTNs were associated with more than one trait. Of these 160 QTNs, 73 were detected in two or more environments and were considered reliable QTLs for the respective traits. A total of 135 associated QTNs were annotated and located within the genes, including ABC transporter, Cytochrome450, Thioredoxin_M-type, and hypothetical proteins. Furthermore, the expression pattern of annotated QTNs demonstrated that only 122 were differentially expressed, suggesting these could potentially be related to seed development. The genomic regions/candidate genes for grain size traits identified in the present study represent valuable genomic resources that can potentially be utilized in the markers-assisted breeding programs to develop high-yielding varieties.

## Introduction

Bread wheat (*Triticum aestivum* L.) is an important staple food crop, serving as the main source of energy, protein, and fiber for much of the world’s human population (Ling et al., 2013; Ji et al., 2022). However, with the current rate of yearly increment in wheat yield, feeding the ever-increasing world population, which is expected to reach 9.3 billion by 2050, will be a daunting task. Further, depletion of natural resources such as land and water and a rise in the mean earth surface temperature exacerbates the problem and poses a challenge to producing sufficient wheat to feed the human population in the future (Nehe et al., 2019). To increase wheat yield, it is important to understand the genetic basis of traits that contribute to grain yield (GY). Grain yield and its contributing traits are complex in nature, highly influenced by environmental conditions, and regulated by multiple genes (Kato et al., 2000; Li et al., 2019; Ji et al., 2022). Quantitative trait loci (QTL) associated with GY has been extensively studied and reported on all 21 wheat chromosomes (Bennett et al., 2012; Sun et al., 2020). Several studies have identified numerous QTL for GY and productivity (Bennett et al., 2012; Zegeye et al., 2014; Malik et al., 2021; Ji et al., 2022). However, there is very limited information on the marker-assisted improvement of GY traits in wheat. This is primarily due to the non-availability of tightly linked robust markers with the GY-associated traits. The conventional QTL mapping approach has been extensively used for gene mapping and has enabled genetic dissection of seed traits in wheat (Breseghello and Sorrells, 2007; Duan et al., 2020). However, this approach does not allow detection of all the possible allelic variants of the target gene that might exist in the natural populations of wheat. Another downside of the QTL mapping approach is its poor resolution. Availability of gold standard wheat reference genome sequence and high-density SNP arrays is expected to accelerate high-resolution mapping of complex traits using both conventional and association mapping approaches (IWGSC et al., 2018; Chaurasia et al., 2021).

In the past few years, the genome-wide association study (GWAS) has become a popular approach to identify the QTL associated with complex traits in crops. In contrast to QTL mapping, this approach enables the exploration of a large number of alleles for any locus from a natural population of diverse individuals. This approach facilitates high-resolution mapping of traits because the individuals used for the association analysis might have undergone several rounds of historical recombination (Yu and Buckler, 2006; Li et al., 2019). Several GWAS studies have been performed on major crops such as *Oryza sativa* (Spindel et al., 2016), *Zea mays* (Xu et al., 2017; Xu et al., 2018), *Hordeum vulgare* (Visioni et al., 2013), *Avena sativa* (Newell et al., 2011), *Brassica napus* (Zhou et al., 2017), *Glycine max* (Zhang et al., 2015), *Sorghum bicolor* (Morris et al., 2013), and in wheat for the genetic dissection of various desirable traits (Peng et al., 2018; Chaurasia et al., 2021; Malik et al., 2021). Over the past few years, efforts have been made to develop GWAS models that are more suited to investigating genetics of simple as well as complex traits in plants. These GWAS models are broadly grouped into single-locus GWAS (SL-GWAS) and multi-locus GWAS (ML-GWAS) methods. SL-GWAS methods have been widely used to detect genetic variants for various traits, but one main limitation of this model is that the p-values of the markers identified to be associated with the target trait need to be subjected to multiple rounds of testing to avoid false positive associations. To overcome this limitation, Zhang et al. (2020) developed a mrMLM package that contains the following six ML-GWAS methodologies: mrMLM (multilocus random-SNP-effect MLM) (Wang et al., 2016), FASTmrMLM (fast mrMLM) (Tamba et al., 2017), ISIS EM-BLASSO (iterative modified-sure independence screening expectation-maximization Bayesian least absolute shrinkage and selection operator) (Wen et al., 2017), pKWmEB (integration of Kruskal-Wallis test with empirical Bayes) (Ren et al., 2018), FASTmrEMMA (fast multi-locus random-SNP-effect efficient mixed model analysis) (Wen et al., 2017), and pLARmEB (polygenic-background-control-based least angle regression plus empirical Bayes) (Zhang et al., 2017).

Among the GY-associated traits, grain size contributes the most, making it a key selection target in wheat breeding programs for developing high yielding varieties. Thousand grain weight (TGW) is the main component of GY and is determined by grain size traits such as grain length (GL), grain width (GW), and grain length width ratio (GLWR) (Sun et al., 2009; Li et al., 2022). Thus, it is important to understand the genetic and molecular basis of the mechanisms governing grain size in wheat genotypes and to identify the superior novel alleles governing this trait from germplasm collections for exploitation in the breeding program. Therefore, the main aim of this study was to dissect the genetic control of grain size traits such as GL, GW, GLWR, and TGW in wheat germplasm employing 35K SNP array using multi-locus GWAS approaches.

## Materials and methods

### Experimental materials and phenotyping

The experimental material for the GWAS study comprised of 125 diverse wheat accessions, a subset of a mini core developed from the composite wheat core set (Phogat et al., 2020) of the National Genebank of India. These accessions were comprised of 85 indigenous and 40 exotic collections, which included released varieties, landraces, genetic stocks, and elite genotypes (**Supplementary Table 1**). These accessions were evaluated following augmented block design in five blocks using four checks (HD2967 and C-306) randomized in each block over five years (*Rabi* 2015-16 to *Rabi* 2019-20). The GWAS panel was evaluated at the ICAR-National Bureau of Plant Genetic Resources (NBPGR), Issapur Farm, Delhi located 28.3748° N, 77.0902° E, 228.6 m AMSL, for five consecutive years; during the fifth year, the trial was also taken up at the ICAR-NBPGR, Regional Station, Jodhpur located at 26.2389° N, 73.0243° E, 263 m AMSL) and the Zonal Agricultural Research Station, Powarkheda, Madhya Pradesh located at 22.4154° N, 77.4442° E, 229 m AMSL. In total, these made up seven environments: Delhi (2015-16)-E_1_, Delhi (2016-17)-E_2_, Delhi (2017-18)-E_3_, Delhi (2018-19)-E_4_, Delhi (2019-20)-E_5_, Powarkheda (2019-20)-E_6_, and Jodhpur (2019–20) - E_7_.

Each genotype was grown in a three-rows plot of 2 m length each, with a row-to-row distance of 0.25 m. Pests and diseases were controlled chemically, whereas weeds were controlled manually. The wheat GWAS panel was evaluated for GL (mm), GW (mm), GLWR, and TGW (gm) from the harvested grain samples. The measurements of these grain parameters were performed by selecting the main spike of five random individual plants in the middle of the row for each accession. Grains per spike were estimated by hand-threshing the mature spike. TGW of each genotype was recorded by weighing all the seeds from a sample, dividing it by the total seed number measured, and multiplying the result by 1000. For GL and GW analysis, ten seeds from each five spikes were measured using digital vernier caliper and average value of the plot accessions was taken up for analysis. Grain length/width ratio (GLWR) was calculated by dividing the grain length mean by the grain width mean for each genotype.

### Phenotypic data analyses

The phenotypic data was analyzed using ACBD-R (Augmented Complete Block Design with R) version 4.0 software (Rodriguez et al., 2018). The mean, coefficient of variation (CV), least significant difference (LSD), genotypic variance, and heritability were estimated. In ACBD-R v4.0, the best linear unbiased predictors (BLUPs) of each genotype were calculated for each environment and across environments along with four checks varieties. The calculated BLUPs were then used in the GWAS analysis. The frequency distribution graphs, correlation coefficients of the recorded traits, and principal component analysis were obtained through SAS JMP Version 14 software (https://www.jmp.com/en_in/software/data-analysis-software.html).

### Genomic DNA isolation and SNP genotyping

Genomic DNA was isolated from one-week-old wheat seedlings using the CTAB method (Murray and Thompson, 1980) with a few modifications and then treated with RNase to remove any RNA contamination. The integrity of DNA samples was checked on 0.8% agarose gel and concentration was determined by using a NanoDrop1000 (Thermo Scientific). Genotyping of isolated DNA samples was done using Breeder’s 35K Axiom^®^ array (Allen et al., 2017). The SNPs with a genotyping call rate < 97% and minor allele frequency (MAF) <5% were removed while performing genomic data analysis.

### Clustering, population structure, and linkage disequilibrium analysis

A total of 23,874 SNPs were used to perform principal component analysis (PCA) and generate kinship matrix using TASSEL 5.2 program (https://www.maizegenetics.net/tassel). STRUCTURE software was used to estimate the level of genetic differentiation in the population using the Bayesian model-based approach; the parameter burn-in period and Monte Carlo Markov Chain (MCMC) replication number were set to 10,000 and 20,000 respectively for ten independent runs to estimate the number of subpopulations (k) in a putative range of k = 1 to 5. The optimal subpopulation number was estimated using an *ad hoc* statistic delta k (Evanno et al., 2005). The squared allele frequency correlation (r^2^) between SNP markers was used to estimate linkage disequilibrium (LD) using TASSEL v5.2 (https://www.maizegenetics.net/tassel).

### Genome-wide association analysis

All 125 accessions were genotyped using 35K SNPs array. We used five ML-GWAS methods which were included in the R package mrMLM v4.0.2 (https://cran.r-project.org/web/packages/mrMLM/index.html). These five models are mrMLM, FASTmrMLM, FASTmrEMMA, pLARmEB, and ISISEM-BLASSO. All the parameters were set at default values. The critical thresholds of significant association for all the five methods were set as logarithm of the odds (LOD) score ≥3.00. The most significant SNPs, detected in at least two methods, were considered as reliable SNPs.

### Differential gene expression analysis

We utilized RNA-seq data of two wheat genotypes with contrasting seed size i.e., IC111905 (large-seeded)and EC575981 (small-seeded) at 15 days and 30 days post anthesis (DPA) with three biological replicates to check the expression profile of putative candidate genes located in the identified genomic regions. Illumina sequencing was performed, which generated approximately 177 Gb raw data. Approximately 98.5% of reads passed the quality control and clean reads were mapped back on to the reference genome (IWGSC v2.0) (https://plants.ensembl.org/Triticum_aestivum/Info/Index) using bwa-mem software (https://sourceforge.net/projects/bio-bwa/files/). The differential gene expression analysis was performed using edge R package and genes with p-value <0.05 were considered as significantly differentially expressed genes. Heat maps of differentially expressed genes were generated by MeV software (https://sourceforge.net/projects/mev-tm4/).

## Result

### Phenotypic evaluation and variability

All the genotypes of the wheat association panel were phenotyped for grain size parameters (GL, GW, GLWR, and TGW) in seven different environments (E_1_-E_7_). The descriptive statistics of the investigated traits in seven environments are presented in **Supplementary Table 2**, and revealed wide variability for all the traits. GL ranged from 5.24 to 8.20 mm in E_1_, 5.09 to 8.17 mm in E_2_, 4.83 to 8.15 mm in E_3,_ 5.04 to 8.09 mm in E_4,_ 4.43 to 7.79 mm in E_5_, 4.43 to 8.41 mm in E_6_, and 4.47 to 7.87 mm in E_7_. GW ranged from 2.29 to 3.95 mm in E_1_, 2.26 to 3.71 mm in E_2_, 1.89 to 3.73 mm in E_3,_ 1.87 to 3.69 mm in E_4,_ 1.41 to 3.67 mm in E_5_, 1.41 to 3.58 mm in E_6_, and 1.48 to 3.81 mm in E_7_. GLWR ranged from 1.49 to 2.65 in E_1_, 1.29 to 2.70 in E_2_, 1.64 to 3.12 in E_3,_ 1.64 to 3.52 in E_4,_ 1.62 to 3.12 in E_5_, 1.69 to 3.12 in E_6,_ and 1.61 to 3.02 in E_7_. TGW ranged from 18.00 to 64.54 in E_1_, 13.51 to 67.56 in E_2_, 7.91 to 51.93 in E_3,_ 9.87 to 61.39 in E_4,_ 12.17 to 56.66 in E_5_, 13.75 to 67.21 in E_6,_ and 14.26 to 55.51 in E_7_. The coefficients of variation for GL, GW, GLWR, and TGW ranged from 8.32% to 24.00%, indicating considerable variability for these traits. The CV percent was highest for TGW in E_6_ (24.00%) followed by E_4_ (21.62%) and E_2_ (20.39%). The frequency distribution of four traits (GL, GW, GLWR, and TGW) (**Supplementary Figure 1**) showed near normal distribution in all environments, indicating the quantitative nature of these traits except for GW under environments E_3_ and E_4_.

Based on BLUP analysis (**Supplementary Table 3**), genotypes recorded an overall grand mean of 6.33 ± 0.40 mm, 6.34 ± 0.39 mm, 6.28 ± 0.36 mm, 6.23 ± 0.44 mm, 6.27 ± 0.34mm, 6.52 ± 0.28 mm, and 6.64 ± 0.63 mm respectively under different environments for grain length, whereas for grain width, overall means of 3.19 mm ± 0.06, 3.19 ± 0.07 mm, 3.10 ± 0.18 mm, 3.02 ± 0.39 mm, 3.22 ± 0.22 mm, 3.08 ± 0.41 mm, and 3.20 ± 0.41 mm respectively were recorded. GLWR recorded overall mean of 2.02 ± 0.40, 2.02 ± 0.40, 2.06 ± 0.15, 2.08 ± 0.16, 1.95 ± 0.15, 2.12 ± 0.14, and 2.13 ± 0.18 in different environments, whereas TGW recorded grand means of 42.60 ± 5.24 g, 44.30 ± 6.73 g, 37.66 ± 3.00 g, 39.85 ± 6.84 g, 43.07 ± 5.08 g, 38.35 ± 5.30 g, and 42.00 ± 3.12g respectively. Promising accessions were identified based on adjusted BLUP mean. The top ten accessions for GL were EC578134 (7.457mm), IC539313 (7.148 mm), EC339611 (7.106 mm), EC464070 (7.085 mm), C697725 (7.036 mm), IC252928 (7.033 mm), IC535217 (7.016 mm), EC542279 (7.014 mm), EC313710(7.005 mm), and EC578152(6.969 mm). For GW, the top ten accessions were IC252429 (3.753 mm), IC335683 (3.441 mm), IC252954 (3.432 mm), IC335715 (3.418 mm), IC252772 (3.38 mm), IC574476 (3.378 mm), IC252422 (3.369 mm), IC75240 (3.355 mm), IC122726(3.349 mm), IC116274(3.34 mm), and IC539314(3.328 mm). Similarly, promising accessions with more than 50 g thousand grain weight were identified as IC539313 (55.03 g), EC578134 (53.56 g), IC542076 (50.95 g), IC335715 (50.36 g), and EC578152 (50.0g).

The environment-wise heritability and variance components based on BLUP value are presented in **Supplementary Table 3**. Heritability for GL ranged from 22.5% (E_7_) to 82.4% (E_4_), whereas heritability for GW ranged from 22.3% (E_7_) to 60.2% (E_4_). Similarly, heritability for GLWR ranged from 22.0% (E_1_) to 62.3% (E_5_), whereas TGW was found to be heritable in the range of 21.8% (E_6_) to 74.1% (E_3_).

### Multivariate analysis

#### Correlation between traits in different environments

Pearson’s correlation coefficients were estimated among grain traits for diverse wheat accessions under each environment separately (**Supplementary Table 4**). GL was found to have a consistently significant positive correlation with GW (0.368, 0.406, 0.444), GLWR (0.585, 0.562, 0.335), and TGW (0.471, 0.394, 0.538) under E_1_, E_2,_ and E_3_ respectively. Contrarily, GW showed a negative correlation with GLWR (-0.363, -0.438, and -0.684) and significant positive correlation with TGW (0.371, 0.380, and 0.604) under E_1_, E_2_, and E_3_ respectively. Under the environment E_4_, GL showed significant positive correlations with GW, GLWR, and TGW that ranged between 0.225 (GW) to 0.457 (GLWR), while GW showed highly a significant negative correlation with GLWR (-0.748) and significant positive correlation with TGW (0.488). Similarly, under E_5_, a significant positive correlation was observed with GW (0.406), GLWR (0.361), and TGW (0.643), while GW showed a significant negative correlation with GLWR (-0.686) and significant positive correlation with TGW (0.723). A similar correlation pattern among seed traits was also observed under E_6_ and E_7_ (**Supplementary Table 4**).

#### Phenotypic correlation between different environments for traits

The magnitudes of correlation between environments were assessed for knowing behavior of genotypes for trait expression. A total of twenty-one combinations of correlations were observed between different pairs of environments for all the traits (**Supplementary Table 5**). Here, significant positive associations were revealed for response of traits by genotypes for all environment pairs except five for grain length, and TGW and three for grain width and GLWR. For GL, these correlation values ranged from 0.084 between E_3_ to E_7_ to 0.985 between E_1_ to both E_2_ and E_4_. For GW, these correlation values ranged from 0.061 between E_4_ to E_7_ to 0.953 between E_1_ and E_2_. Similarly, for GLWR, the lowest association was observed as 0.089 between E_4_ to E_6_ and highest as 0.961 between E_1_ and E_2_. For TGW, lowest association was observed as 0.021 between E_4_ to E_6_ and highest as 0.954 between E_2_ and E_4_.

#### Principal component analysis and correlation study based on pooled analysis

**Figure 1:**
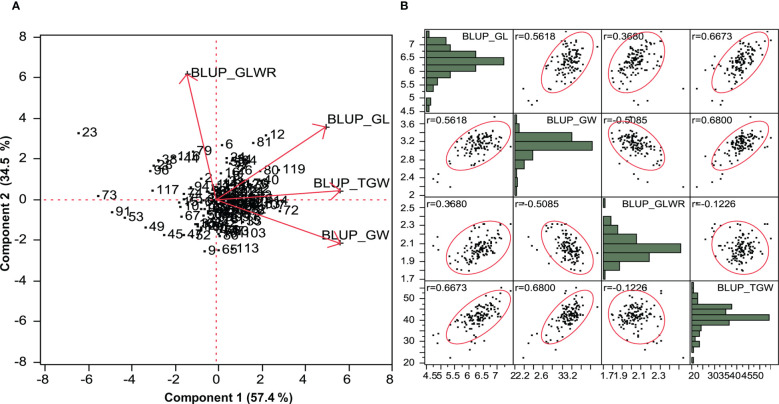
**(A)** Principal component biplot based on BLUP value of grain parameters over the environments. **(B)** Scatter plot showing correlation matrix between grain parameters based on BLUP value.

PCA was performed on the basis of pooled data for seven environments for grain parameters. Genotype by trait biplot depicted two-dimensional spatial diversity of accessions as well as trait variability (**Figure 1A**). High trait variability was observed for traits like GLWR, GL, GW, and TGW, which was evidenced by the larger length of the characters and high positive correlations between traits such as GL and TGW and GLWR and GL, and a negative correlation between TGW and GW, which is evident by the narrow angle between them. Here, the first principal component, PC1, explained 57.4% of the cumulative variation. The major contributing traits for PC1 were GW (0.90), TGW (0.90), and GL (0.79) in positive direction. Second principal component, PC2, explained 34.5% of the cumulative variation. The major contributing traits for PC2 were GLWR (0.97) and GL (0.56). Further, we analyzed correlations between traits using the BLUP values where GL showed a highly significant positive correlation with GW (0.562), GLWR (0.368), and TGW (0.667), whereas GW showed a highly significant negative correlation with GLWR (-0.508) and significant positive correlation with TGW (0.680). GLWR was not significantly related to TGW (**Figure 1B**).

### Genotyping

**Figure 2:**
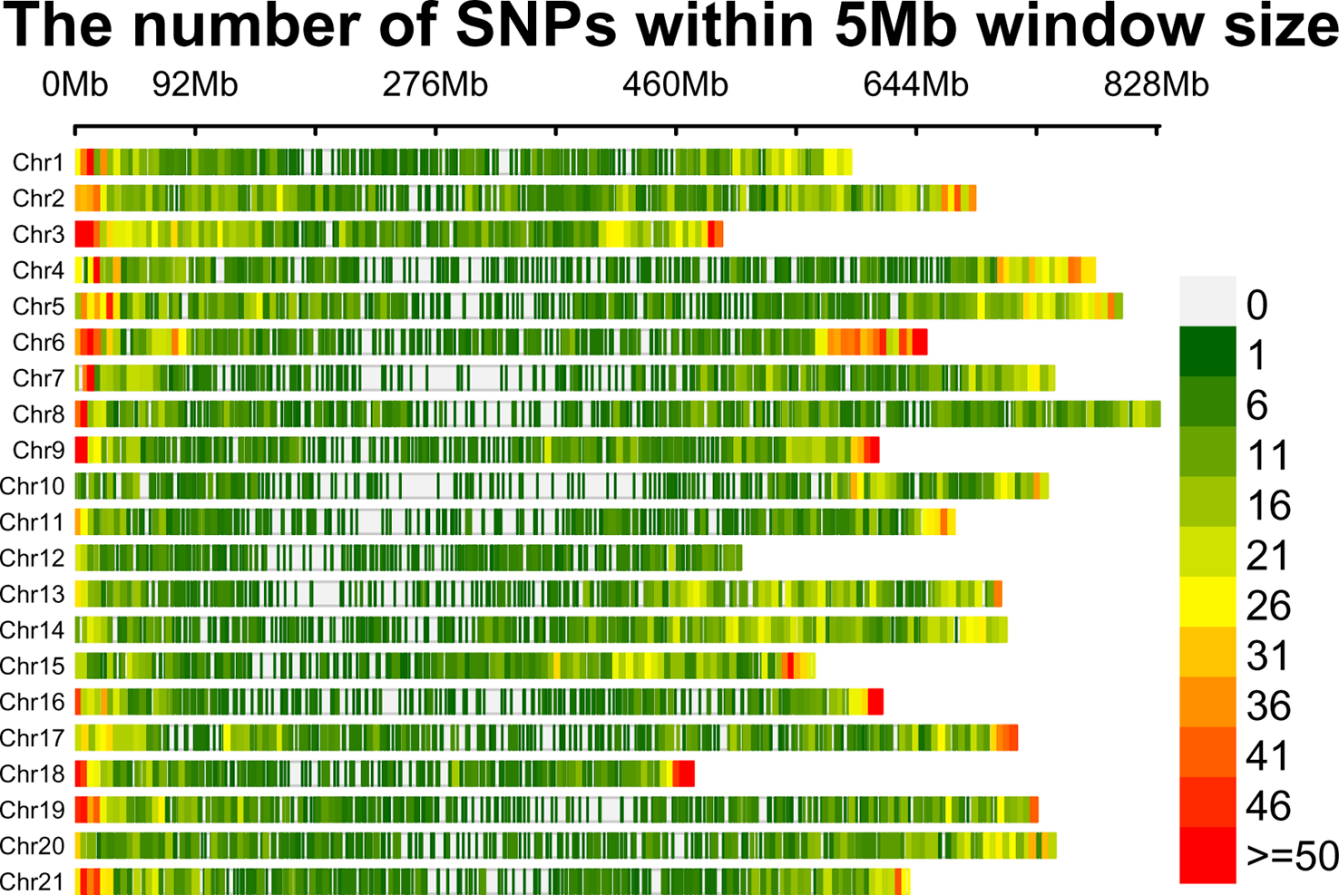
SNP density plot of 21 wheat chromosomes displaying distribution of SNPs within 5 Mb window size. The horizontal axis shows chromosome length (Mb); Different colors depict SNP density.

A total of 125 wheat accessions representing a subset of the Indian National Genbank mini core germplasm were genotyped using 35K wheat SNP array that contains 35,143 genome-wide single nucleotide polymorphism (SNP) markers. The SNP probe sequences of wheat array were BLASTn (https://blast.ncbi.nlm.nih.gov/doc/blast-help/downloadblastdata.html) against wheat genome to find out their physical location, which revealed only 31,926 SNPs with known positions. Furthermore, SNPs were also filtered on the basis of minor allele frequency (≥0.05), missing threshold of < 10%, and call rate ≥97%. Finally, a total of 23,874 SNPs were retained for genetic diversity, population structure, and GWAS analysis. Mapping of 23,874 filtered SNP markers provided a whole genome-wide coverage along the 21 chromosomes of wheat (**Figure 2**). Further, distribution analysis of SNPs on wheat chromosomes revealed that the maximum number of SNPs was positioned on 1B (1594), followed by 2D (1575) and 1D (1516), while the lowest number was positioned on 4D (508), followed by 4B (800) and 4A (808). We also compared the distribution of SNPs on the three wheat sub genomes; it was found that 7,291 SNPs belonged to A sub-genome, 8,784 SNPs were found on B sub-genome, and 7,799 SNPs belonged to D sub-genome (**Table 1**).

**Table 1:** Chromosome-wise distribution of 23,874 SNPs and the intra-chromosomal estimated LD among 125 wheat genotypes.

Chrom	Size (Mb)	No. of SNP	SNP/Mb Density	SNP pair in LD (p <0.05)	SNP pair In LD (r2 = 1)	Average LD (r2)	D prime
**1A**	594.1	1138	1.92	102600	5766	0.220567146	0.71973128
**1B**	689.85	1594	2.31	236002	22116	0.271350626	0.77589076
**1D**	495.45	1516	3.06	163630	19467	0.314246556	0.77693029
**2A**	780.8	1187	1.52	101708	1958	0.189312876	0.66675889
**2B**	801.26	1513	1.89	155261	2648	0.169167472	0.64845702
**2D**	651.85	1575	2.42	146171	6944	0.249166202	0.73375966
**3A**	750.84	1024	1.36	62572	1342	0.17548539	0.66747653
**3B**	830.83	1195	1.44	101455	1207	0.163443076	0.64468071
**3D**	615.55	1158	1.88	62878	3762	0.239489328	0.71345541
**4A**	744.59	808	1.09	39406	685	0.177018377	0.65255529
**4B**	673.62	800	1.19	40467	1107	0.218309804	0.68785027
**4D**	509.86	508	1	12770	1324	0.308622143	0.78044746
**5A**	709.77	1132	1.59	76483	1872	0.171701946	0.64123409
**5B**	713.15	1278	1.79	130839	2647	0.163887549	0.63806885
**5D**	566.08	1113	1.97	72678	4467	0.232972965	0.7229823
**6A**	618.08	845	1.37	43714	1187	0.191258087	0.66474072
**6B**	720.99	1259	1.75	126269	1264	0.171017301	0.65574685
**6D**	473.59	851	1.8	42753	2005	0.217027812	0.70667145
**7A**	736.71	1157	1.57	94614	1230	0.165787476	0.65314744
**7B**	750.62	1145	1.53	78061	1132	0.179186058	0.66074305
**7D**	638.69	1078	1.69	61139	2465	0.237260494	0.72331864

### Population structure, kinship, and linkage disequilibrium decay analyses

**Figure 3:**
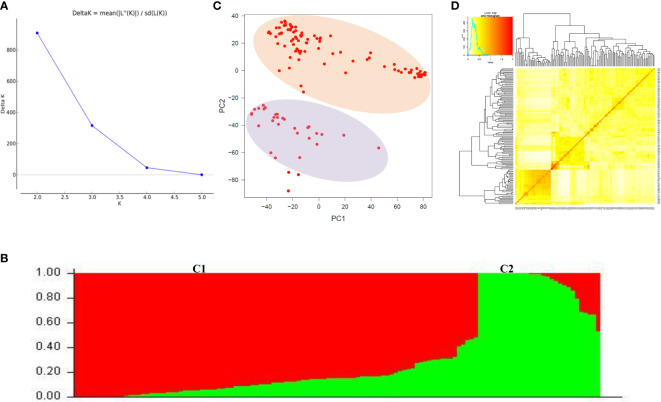
Population structure analysis of wheat association mapping panel. **(A)** Magnitude of ΔK values, rate of change from 2 to 5 in association mapping panel. **(B)** Population structure of association panel based on 125 germplasm-based SNP markers at K = 2. Different color columns represent different sub sub-populations. **(C)** Principal component analysis showing two sub sub-populations. **(D)** Heat map of kinship matrix. The heat map shows the level of relatedness among the population. The darker areas show the level of relatedness between genotypes and the dendrogram depicts clustering of sub sub-population.

We used 23, 874 SNP markers to ascertain the population structure in the wheat mini core set using STRUCTURE and PCA analysis. The most probable number of populations were estimated using delta K method implemented in the STRUCTURE HARVESTOR program. The value of ΔK peaked at K=2 and revealed two sub-populations in the wheat mini core germplasm. Sub-population 1 represented 82% of the individuals; out of that, 62% were pure and 38% admixtures. Whereas sub-population 2 had 18% of the individuals of the AM panel, and contained 75% pure and 25% admixtures. PCA also detected the two sub-populations indicated by two significant components, explaining the maximum variation of the population. Further, kinship matrix was also created to explore the relationship among the individuals using the genome association and prediction integrated tool (GAPIT) which demonstrated the presence of two sub-groups within the association panel (**Figure 3**).

**Figure 4:**
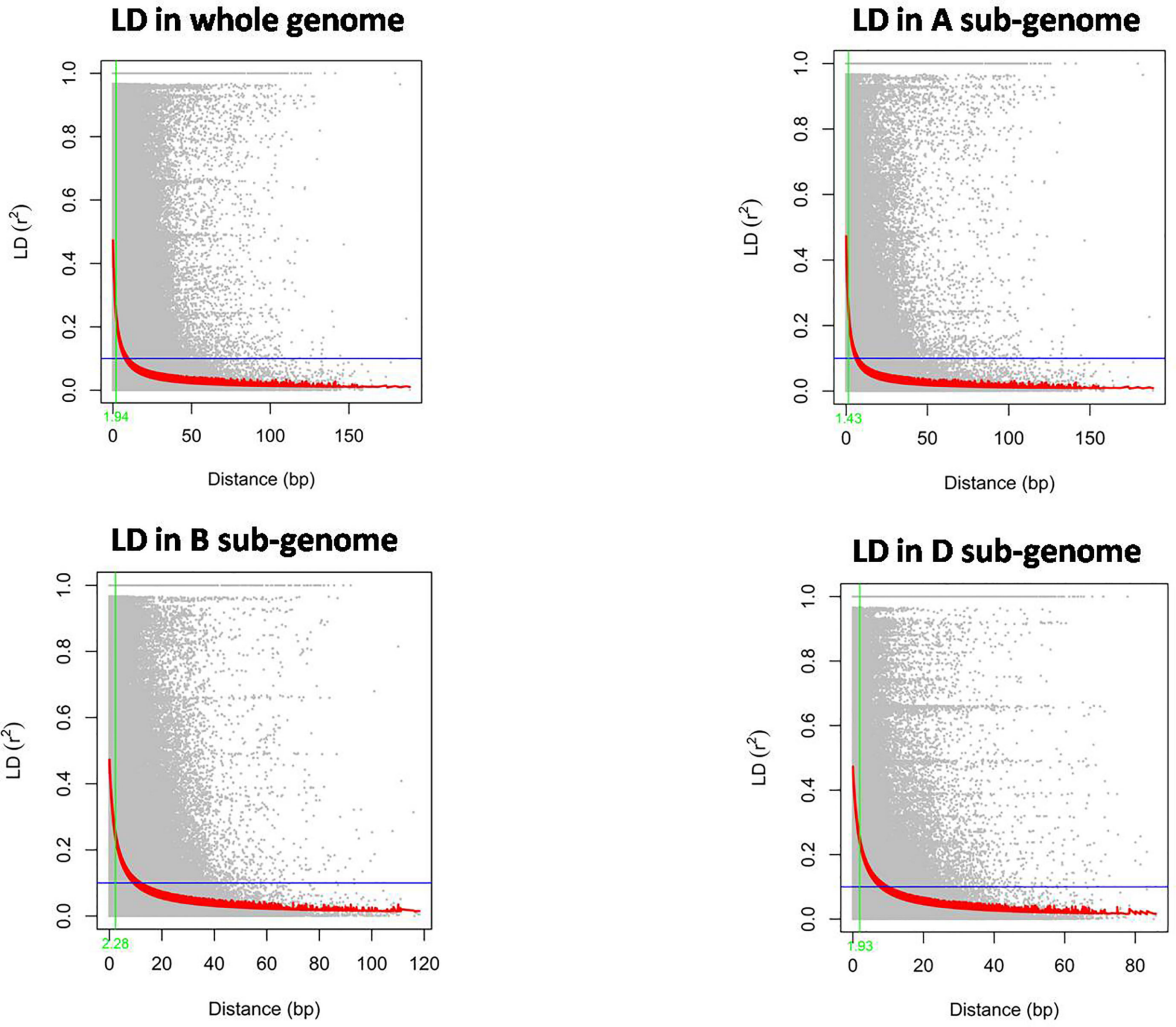
The rate of Linkage disequilibrium decay (R^2^) between pairs of polymorphic markers of the whole wheat genome and its sub-genomes A, B, and D are plotted against the genetic distance (Mb).

The LD decay in the wheat mini core set was estimated by calculating the squared correlation coefficient (r^2^) for all the SNPs. The LD decay for the whole genome was 1.9 Mb. Further, it was found that the decay was most rapid in the A sub-genome (1.63 Mb), followed by the D sub-genome (1.93 Mb) and B sub-genome (2.28 Mb) (**Figure 4**).

### GWAS for grain size traits

**Figure 5:**
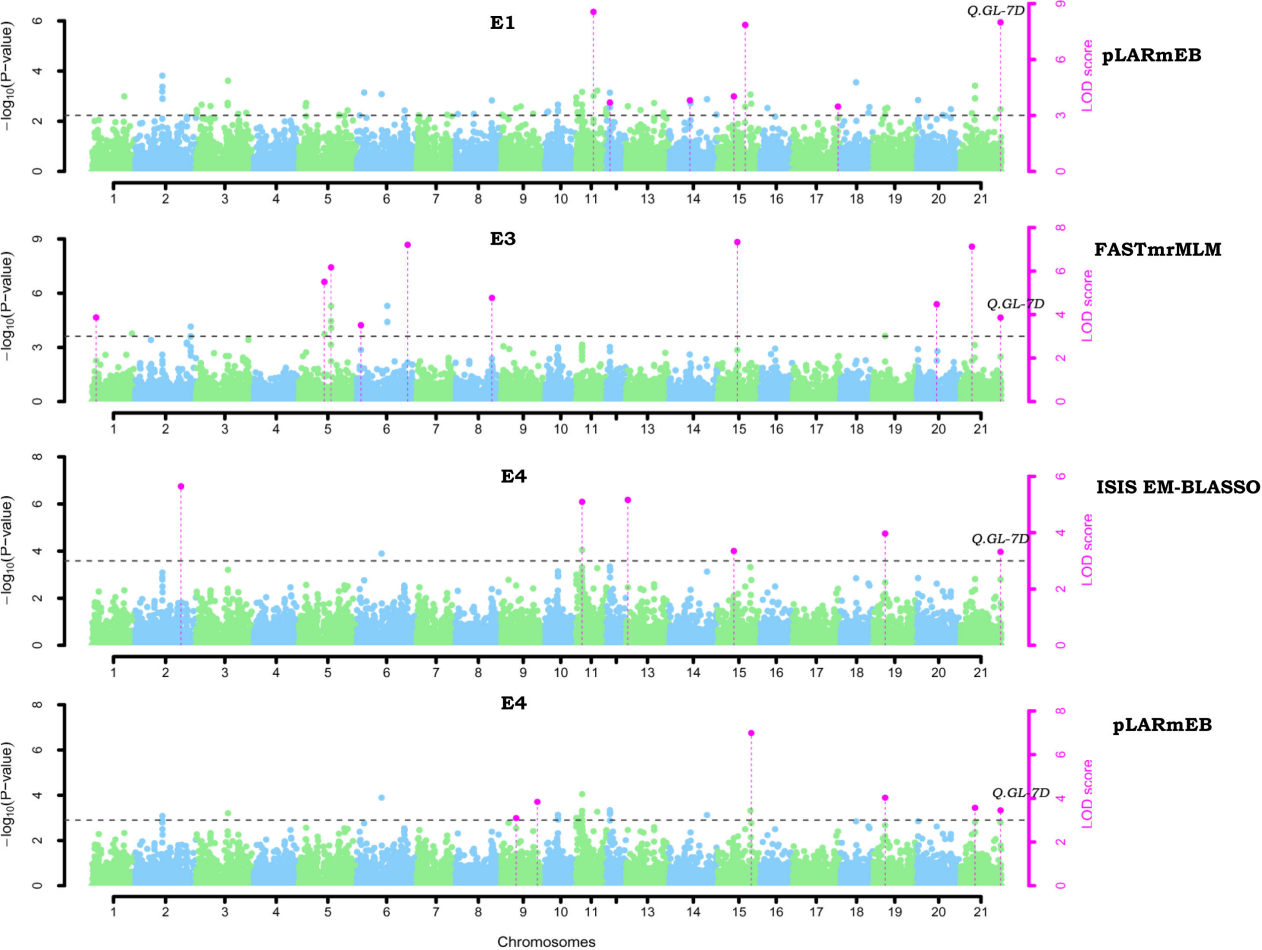
Manhattan plots of associated QTNs for grain length (GL) in wheat using multi-locus GWAS model. The x-axis shows the chromosome label and the y-axis displays - thresholds for significance (LOD score = 3) and log10 (p-value). The significant QTNs with LOD score >=3 is represented with purple dots.

**Figure 6:**
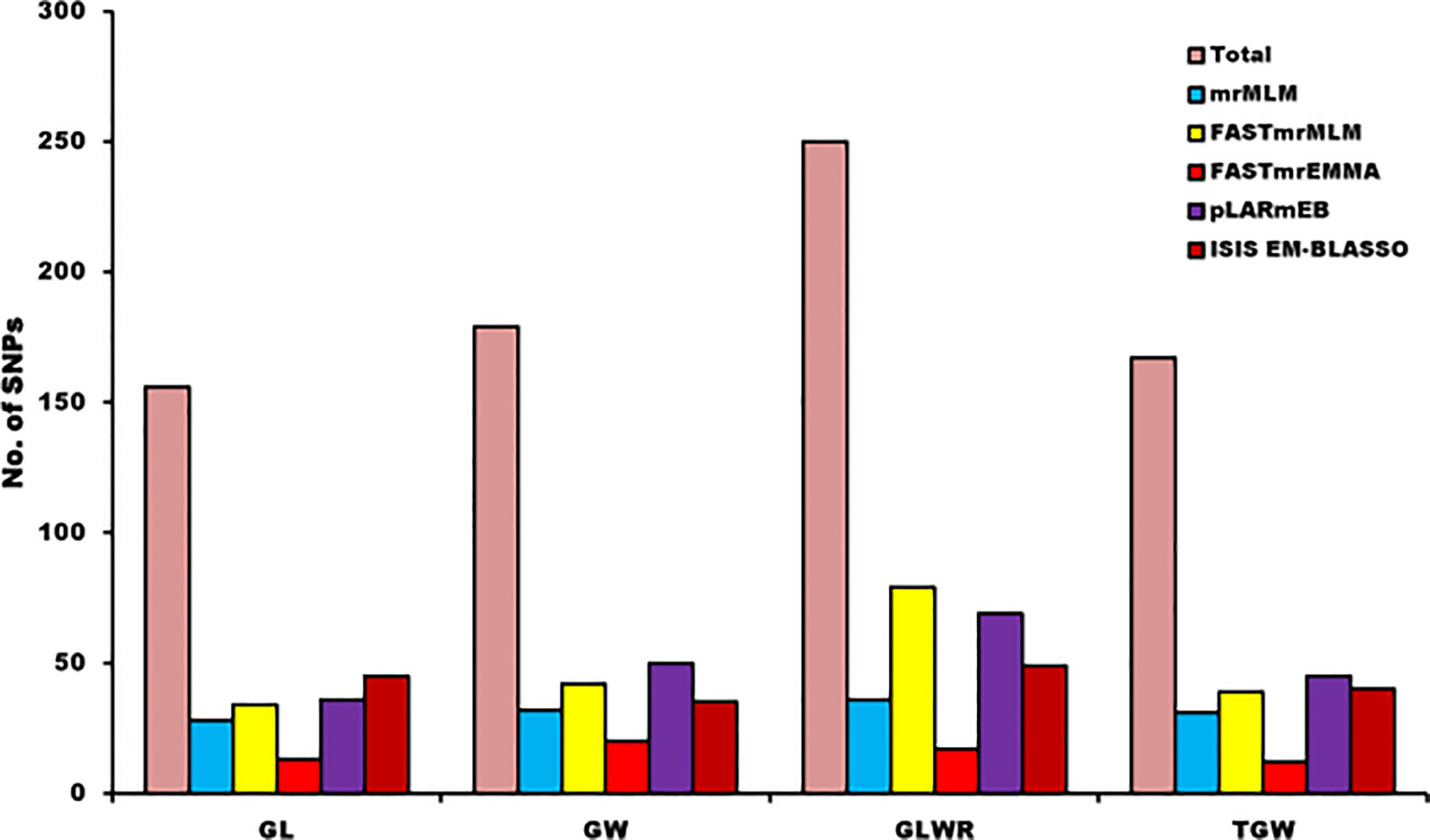
Distribution of identified and significant SNPs for each trait on the basis of detection models of multi-GWAS.

GWAS was performed using 23,874 SNPs filtered on various parameters to identify genomic loci associated to four different grain yield traits (GL, GW, GLWR, and TGW) independently for the seven environments and also based on the BLUP values derived from data of grain size traits of all the seven environments. Here, we have used five multi-locus models (mrMLM, FASTmrMLM, FASTmrEMMA, pLARmEB, and ISIS EM-BLASSO) to conduct GWAS. A total of 752 significant SNPs were predicted for four grain size traits using ML-GWAS models with LOD score ≥ 3. Manhattan plots for GL drawn using various ML-GWAS models that depict marker trait associations are presented in **Figure 5**. Of these 752 SNPs, 72 were identified using BLUP values derived from the data of all the environments and other SNPs were identified by analyzing data of each location separately. We classified 752 SNPs according to trait, which demonstrated that 156, 179, 250, and 167 SNPs were associated with GL, GW, GLWR and TGW traits, respectively (**Figure 6**). In addition, comparative study on the basis of the GWAS model demonstrated that 28, 32, 36, and 31 SNPs were predicted for four traits (GL, GW, GLWR, and TGW respectively) by the mrMLM model while the FASTmrMLM model could detect 34, 42, 79, and 39 SNPs for GL, GW, GLWR, and TGW. FASTmrEMMA and pLARmEB model identified 13, 20, 17, and 12 and 36, 50, 69, and 45 SNPs for the four traits (GL, GW, GLWR, and TGW respectively). The ISIS EM-BASSO model detected 45, 38, 49, and 40 SNPs for GL, GW, GLWR, and TGW respectively (**Figure 6**).

On the basis of redundancy of SNPs in the models and locations, we combined the identified SNPs and found a total of 160 SNPs were simultaneously detected in two or more multi-locus models. These SNPs were designated as reliable *QTNs* for the respective traits. Furthermore, distribution of these 160 significant SNPs was also analyzed across the environments. Out of these 160 *QTNs*, 87 were confined to only one environment that included 13, 19, 27, and 25 QTNs for GL, GW, GLWR, and TGW respectively, and 3 *QTNs* that were associated with more than one trait (**Table S6**). On the other hand, a total of 73 *QTNs* were simultaneously identified in two or more environments as well as two or more models (**Table 2**). Among these 14, 17, 11, and 10 *QTNs* were identified for GL, GW, GLWR, and TGW traits respectively while 21 SNPs were associated with more than one trait. The physical distribution of all the 160 SNPs on chromosomes demonstrated that SNPs were present on all the chromosomes. Moreover, the highest number of SNPs were found on chr3D (7 SNPs), followed by chr2D (6 SNPs), and chr7D (6 SNPs) while chr1D, chr6B, and chr6D had only one SNPs.

**Table 2:** List of significant QTNs detected simultaneously using two or more multi-locus GWAS methods for four wheat yield-related grain shapes across the environments.

QTN	SNP	Traits	Location	Chr	Position	LOD	R^2^	Models
**Q.GW-1A**	AX-94592848	GW	E_1_,E_2_	chr1A	3.34E+08	5.69~5.88	0.92~2.1	4,2
**Q.TGW-1A**	AX-94932678	TGW	E_2_,E_4_	chr1A	5.58E+08	3.18~8.8	11.48~23.69	1,2,4,5,5
**Q.GW-1A**	AX-94997333	GW	E_1_,E_2_	chr1A	20094503	4~18.51	6.49~23.71	1,2,4,5,4
**Q.TGW-GW-1A**	AX-95120969	TGW,GW	E_1_,E_6_	chr1A	3.38E+08	3.04~5.3	5.93~6.16	5,3
**Q.GLWR-1A**	AX-95213485	GLWR	E_1_,E_3_	chr1A	25708710	3.12~26.68	3.32~33.74	1,2,4,5,4,5
**Q.TGW-1B**	AX-94517103	TGW	E_2_,E_4_	chr1B	6.41E+08	3.87~9.84	7.95~8.58	3,4
**Q.GL-1B**	AX-94699549	GL	E_1_,E_4_	chr1B	5.85E+08	4.17~5.65	3.05~6.42	5,5
**Q.TGW-1B**	AX-94830564	TGW	E_2_,E_4_	chr1B	74228850	4.11~10.66	7.94~14.14	4,1,2,5
**Q.GW-GLWR-1D**	AX-95098685	GW,GLWR	E_3_,E_4_	chr1D	4.17E+08	4.28~5.67	6.66~10.8	1,2,1,4,5
**Q.GW-2A**	AX-94402160	GW	E_1_,E_2_	chr2A	1.92E+08	3.71~10.65	0~9.68	3,4,2,3,4
**Q.GLWR-2A**	AX-94736090	GLWR	E_1_,E_2_, E_3_,E_4_,E_5_	chr2A	7.69E+08	3.26~5.91	4~11.47	1,4,5,5,1,2,4,5,3
**Q.TGW-2A**	AX-94780053	TGW	E_2_,E_4_	chr2A	7.79E+08	3.19~7.47	3.12~14.19	1,2,4,5,5
**Q.TGW-2A**	AX-95012027	TGW	E_2_,E_4_	chr2A	71666146	6.22~6.6	4.77~7.37	4,5
**Q.TGW-GW-2B**	AX-94470912	TGW,GW	E_3_,E_5_	chr2B	7.73E+08	3.85~5.26	9.44~14.2	2,3
**Q.GW-2B**	AX-94519462	GW	E_3_,E_4_	chr2B	5.46E+08	3.87~6.47	7.7~12.23	1,2,3,1,2,3
**Q.GL-GW-2B**	AX-94878848	GL,GW	E_3_,E_4_	chr2B	5.46E+08	5.66~6.67	7.97~17.37	1,2,4,5,4,5
**Q.GLWR-GL-2B**	AX-95129853	GLWR,GL	E_1_,E_3_	chr2B	3.26E+08	4.54~8.2	3.71~28.72	2,4,1,2,4,5
**Q.GL-TGW-2D**	AX-94618441	GL,TGW	E_3_,E_4_	chr2D	6.23E+08	3.32~7.21	7.41~12.87	2,1,5
**Q.GW-2D**	AX-94655905	GW	E_1_,E_2_	chr2D	96623607	4.23~7.86	0~2.05	4,2,4
**Q.TGW-2D**	AX-94745278	TGW	E_2_,E_4_	chr2D	22451354	3.32~10.06	3.8~11.63	1,2,4,5,1,2,5
**Q.GLWR-2D**	AX-94774424	GLWR	E_3_,E_4_	chr2D	4.85E+08	4.47~11.69	3.06~6.63	2,5,4,1
**Q.GLWR-2D**	AX-94922377	GLWR	E_1_,E_2_	chr2D	2693668	3.61~4.63	0~23.04	4,1,2
**Q.GL-2D**	AX-95128254	GL	E_1_,E_2_	chr2D		3.52~5.94	2.52~9.48	1,2,5,5
**Q.GW-3A**	AX-95109402	GW	E_1_,E_2_	chr3A	25939226	4.3~5.83	0.95~3.02	4,5,4
**Q.GL-GL-GL-3A**	AX-95187884	GL	E_1_,E_4_	chr3A	10884903	3.3~4.97	0~12.07	2,1,2
**Q.GLWR–3B**	AX-94671460	GLWR,	E_3_,E_4_	chr3B	7.46E+08	3.47~5.4	0~2.63	4,5,5
**Q.GLWR-3B**	AX-94799334	GLWR	E_1_,E_2_	chr3B	8.18E+08	4.09~4.32	0~6.96	4,2
**Q.GLWR-3B**	AX-95150002	GLWR	E_1_,E_2_	chr3B		5.52~6.85	0.54~7.19	4,4,5
**Q.GW-TGW-3D**	AX-94401378	GW,TGW	E_2_,E_4_	chr3D	6.08E+08	4.24~5.96	4.76~7.65	5,2,4,1
**Q.TGW-3D**	AX-94406908	TGW	E_2_,E_3_,E_4_	chr3D	2.39E+08	3.16~8.2	7.21~11.48	1,2,2,4
**Q.GLWR-GW-3D**	AX-94535556	GLWR,GW	E_1_,E_2_,E_5_	chr3D		3.16~10.15	3.21~7.93	3,4,4,5,1,2,3
**Q.GW-3D**	AX-94642652	GW	E_1_,E_2_	chr3D	1253893	3.33~10.06	10.18~29.66	5,1,4,5
**Q.GW-GLWR-3D**	AX-94749865	GW,GLWR	E_3_,E_4_	chr3D	5.28E+08	4.1~6.7	4.64~21.47	1,4,5
**Q.GL-GW-3D**	AX-95008504	GL,GW	E_1_,E_2_,E_4_,E_5_	chr3D	1.51E+08	3.05~6.33	4.09~14.97	1,2,1,2,4,5,4,5
**Q.GL-GL-3D**	AX-95074739	GL	E_2_,E_4_	chr3D	5.77E+08	3.84~4.85	2.14~2.66	4,4
**Q.GL-4A**	AX-94839917	GL	E_1_,E_2_	chr4A	45529997	4.92~6.13	15.33~31.94	1,2,5,4,5
**Q.GLWR-4A**	AX-95019395	GLWR	E_1_,E_2_	chr4A	29453952	3.6~8.31	2.61~8.88	2,3,5,3
**Q.TGW-4B**	AX-94425015	TGW	E_1_,E_4_	chr4B	2036666	3.2~3.75	1.83~2.94	5,5
**Q.GW-4B**	AX-94433424	GW	E_1_,E_2_	chr4B	6.72E+08	3.78~8.49	0.62~11.76	1,2,4,4
**Q.TGW-GW-GL-4B**	AX-94878781	TGW,GW,GL	E_2_,E_3_,E_5_	chr4B	6.45E+08	3.6~4.86	8.89~16.14	5,1,2,4
**Q.GL -GLWR-4B**	AX-94879134	GL,GLWR	E_1_,E_2_,E_3_,E_4_	chr4B	63231309	3.52~8.12	3.44~7.27	5,3,24,
**Q.TGW-4B**	AX-95232992	TGW	E_1_,E_2_	chr4B	3.89E+08	4.39~5.06	7.48~9.31	3,3
**Q.GW-5A**	AX-94657794	GW	E_3_,E_4_	chr5A	6.74E+08	3.48~4.01	3.53~3.62	4,4
**Q.GL-GW-5A**	AX-94909932	GL,GW	E_1_,E_4_	chr5A	11068517	3.17~9.9	1.54~5.26	5,1,2,4,5,5
**Q.GW-5B**	AX-94424550	GW	E_1_,E_2_	chr5B	4.76E+08	3.42~11.54	1.49~4.19	2,3,4
**Q.GW-5B**	AX-94531833	GW	E_1_,E_2_	chr5B	7.13E+08	3.5~9.78	0~6.7	2,4,5,1,2
**Q.GW-GL-5B**	AX-94711368	GW,GL	E_5_,E_7_	chr5B	7034159	3.71~3.72	2.93~4.95	4,5
**Q.GLWR-5B**	AX-94915493	GLWR	E_1_,E_2_	chr5B	7.03E+08	4.19~18.5	2.94~5.97	2,4,5,1,2,4
**Q.GL-TGW-5B**	AX-95189661	GL,TGW	E_1_,E_3_	chr5B	4.64E+08	3.61~5.05	4.61~12.21	4,2,4
**Q.GL-5D**	AX-94424746	GL	E_1_,E_4_	chr5D	3.54E+08	3.35~4.94	7.6~13.92	4,1,2,5
**Q.GL-5D**	AX-94482861	GL	E_1_,E_2_,E_4_	chr5D	5.15E+08	4.91~10.32	7.8~12.41	1,2,4,4
**Q.GL-5D**	AX-95020206	GL	E_1_,E_2_,E_4_	chr5D	5.02E+08	3.57~4.95	6.38~11.63	3,2,3,2,3
**Q.GL-5D**	AX-95078562	GL	E_2_,E_3_	chr5D	3.85E+08	3.92~7.33	2.37~9.56	5,1,2,3,4,5
**Q.GW-5D**	AX-95192563	GW	E_3_,E_4_	chr5D	3.82E+08	3.29~3.46	0~11.47	2,2
**Q.GLWR-6A**	AX-94615640	GLWR	E_3_,E_4_	chr6A	9253732	5.03~6.88	6.04~6.41	3,2
**Q.GLWR-6A**	AX-94722285	GLWR	E_3_,E_4_	chr6A	3.08E+08	3.47~7.59	5.03~18.87	3,2,4,5
**Q.GW-6A**	AX-95151036	GW	E_1_,E_2_	chr6A	6.00E+08	3.07~3.11	4.49~4.79	3,3
**Q.GL-TGW-GL-6A**	AX-95238912	GL,TGW,GL	E_3_,E_4_,E_5_	chr6A	3.63E+08	3.46~8.23	2.6~23.8	4,4,1,2,3,4,5
**Q.TGW-6A**	AX-95240001	TGW	E_2_,E_4_	chr6A	1.61E+08	3.06~6.9	2.32~5.5	4,2,3
**Q.GL–6B**	AX-94405863	GL,	E_6_,E_7_	chr6B	5.39E+08	5.44~7.24	7.15~13.4	1,2,4,5,4,5
**Q.GW-6D**	AX-95143327	GW	E_3_,E_4_	chr6D	7088328	3.16~4.01	3.9~7.26	3,5,3,4
**Q.GLWR-GL-7A**	AX-94820170	GLWR,GL	E_1_,E_3_	chr7A	68087433	3.26~5.69	0.56~9.02	2,4,4,5
**Q.GL-7A**	AX-95130728	GL	E_2_,E_4_	chr7A	68861347	3.97~6.44	4.52~7.2	4,4,5
**Q.GLWR-GL-7B**	AX-94551830	GLWR,GL	E_1_,E_2_	chr7B	6180054	3.89~7.01	0~4.27	2,5
**Q.GLWR-7B**	AX-94754235	GLWR	E_1_,E_2_	chr7B	6.26E+08	3.81~5.17	2.72~8.3	3,4,5
**Q.GLWR-GL-7B**	AX-94928980	GLWR,GL	E_1_,E_3_	chr7B	6153426	3.62~4.26	0~5.2	2,5
**Q.GL-7B**	AX-95181207	GL	E_1_,E_2_	chr7B	6.88E+08	3.28~5.79	3.54~9.82	1,3,5,5
**Q.GW-GLWR-7D**	AX-94460120	GW,GLWR	E_1_,E_2_	chr7D		3.48~7.27	0.69~10.14	4,3,3,4,5
**Q.GW-7D**	AX-94674756	GW	E_1_,E_5_	chr7D	5.78E+08	3.44~3.81	1.63~6.26	1,4,5
**Q.GL-7D**	AX-94872194	GL	E_1_,E_3_,E_4_	chr7D	6.34E+08	3.32~8	4.82~21.88	4,2,4,5
**Q.GW-7D**	AX-95009696	GW,GW	E_3_,E_4_	chr7D	4.72E+08	3.56~4.04	2.05~3.55	2,5
**Q.GL-7D**	AX-95014664	GL	E_1_,E_2_,E_4_	chr7D	1.03E+08	3.22~6.76	5.76~8.2	2,4,1,2,4
**Q.GW-7D**	AX-95127613	GW	E_3_,E_4_	chr7D	1.01E+08	5.45~6.53	8.82~9.18	3,3

1, mrMLM; 2, FASTmrMLM; 3, FASTmrEMMA; 4, pLARmEB; 5, ISIS EM-BLASSO.

### Allelic effects of identified genomic regions on grain shape

**Figure 7:**
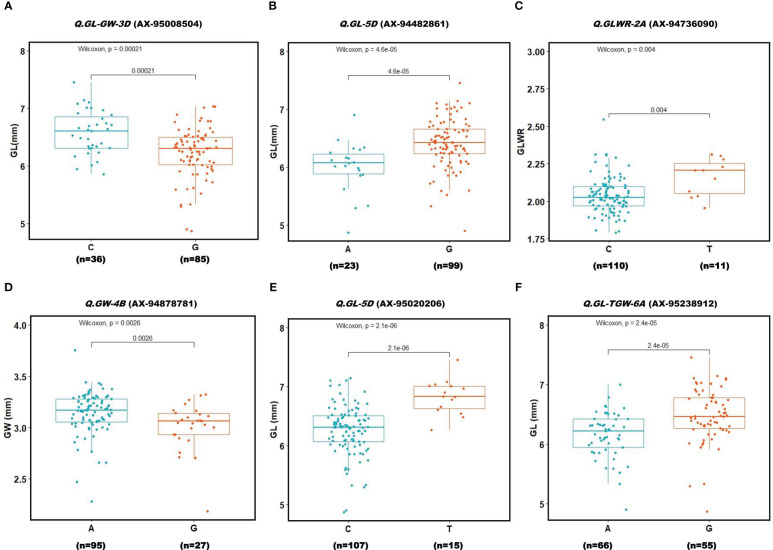
Boxplot for 6 reliable QTNs **(A–F)**. Genotypes were divided into two groups at each locus based on the allele type. A significant difference between the phenotype of these two groups was analyzed using t-test (P ≤ 0.001). Two alleles for each QTN (Locus) are given on X-axis. Y-axis shows phenotypic values of the traits.

To evaluate the allelic effects of *QTNs* on respective phenotype, we only analyzed those *QTNs* that were detected in more than two environments and revealed R^2^ value ≥10% with at least one GWAS model. Association panel genotypes were divided into two groups according to allele types in order to test whether the mean phenotypes of the two groups were significantly different (**Figure 7**). Results showed that six *QTNs* had significant effect (P ≤ 0.01) on their respective traits. Among these six *QTNs*, four QTNs (*Q.GL-GW-3D* (AX-95008504), *Q.GL-5D* (AX-94482861), *Q.GL-5D* (AX-95020206), and *Q.GL-TGW-6A* (AX-95238912)) demonstrated significant effects on GL(mm) whereas one, *QTN Q.GW-4B* (AX-94878781), had significant effect on GW and another, *Q.GLWR-2A* (AX-94736090), showed significant effect on GLWR. The QTNs with significant phenotypic effects on seed traits might contribute to their genetic variations.

### Annotation of identified QTNs

All the 160 significantly associated QTNs with grain size traits that were detected in two or more models were searched for their annotation in the wheat reference genome assembly cv. Chinese Spring (IWGSCrefseq version 2.0, https://wheat.pw.usda.gov/GG3/iwgsc-2.0), available at Plant Ensemble. Of these 160 QTNs, annotation was only detected for 136 QTNs. The detailed analysis of annotated SNPs showed that *Q.TGW-5D* (SNP-AX-95234313) was located within a gene encoding cytochrome 450 and was identified in E4 environment using mrMLM and FASTmrMLM models with LOD score ranging between 3.8 to 3.92. Another, *Q.GW-3D* (SNP-AX-94540502), for grain weight identified at the E6 environment was annotated as ABC transporter and detected by mrMLM and ISIS EM-BLASSO models. We also checked the annotation of QTNs that were identified at multiple environments and found that *Q.GL-1B* (SNP-AX-94699549), *Q.GL-GLWR-4B* (SNP-AX-94879134), *Q.GL-3D* (SNP-AX-95074739), and *Q.GW-5A* (SNP-AX-94657794) were located within genes encoding ABC transporter, WRKY transcription_factor, Glucan endo-1,3-beta-glucosidase, and Zinc_finger_protein respectively. Among these four QTLs, *Q.GL-1B* (encoding for an ABC transporter) was located at 585,331,222bp on chromosome 1B. It was identified in two different environments, E_1_ and E_4,_ using ISIS EM-BLASSO model with LOD scores 4.17 and 5.65 and R^2^ 3.05% and 6.42%, respectively. *Q.GL-GLWR-4B* (encoding for a WRKY transcription factor) was located on chr4B at 63,231,309bp position and identified in four different environments: E_1_ with ISIS EM-BLASSO model, E_2_ with ISIS EM-BLASSO and FASTmrEMMA models, E_3_ with pLARmEB model, and E_4_ with ISIS EM-BLASSO model. It had an LOD score ranging between 3.52 to 8.12. This QTN was associated with GL in all locations except E_2_. Further, in E_2_, this *Q.GL-GLWR-4B* was also determined by three different models, namely FASTmrMLM, pLARmEB, and ISIS EM-BLASSO, but associated with GLWR trait.

### Expression analysis

The transcriptome sequencing of contrasting seed size wheat genotypes, i.e. IC111905 (large-seeded) and EC575981(small-seeded), was performed at two time intervals during the seed development (i.e., 15 and 30 DPA) to quantify expression of all annotated genes within associated genomic regions.

**Figure 8:**
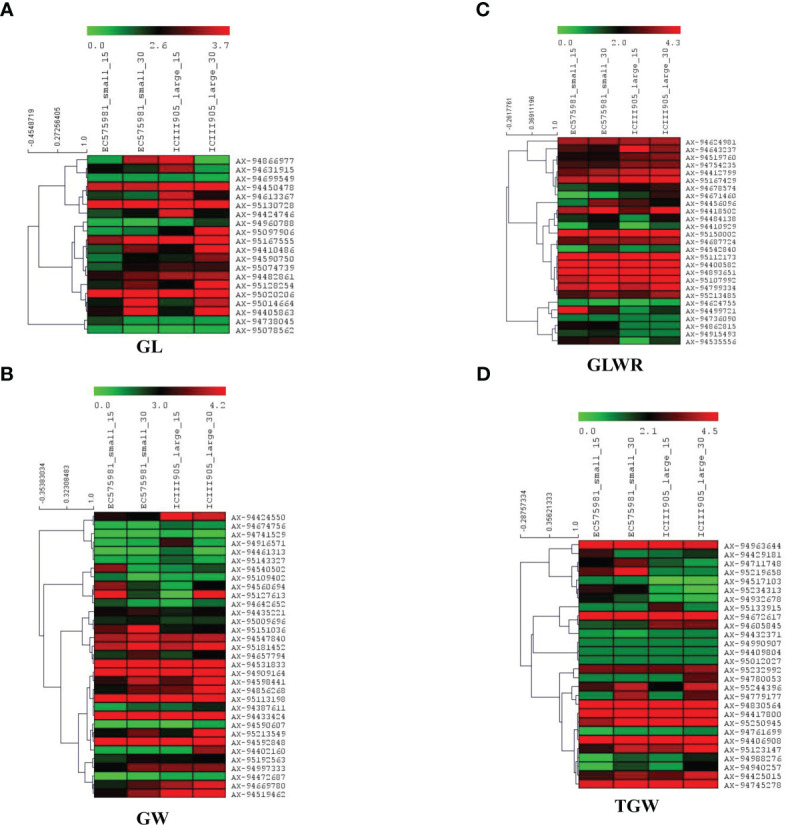
Heat maps of candidate genes identified for four-grain shape traits in small and large-size seeded wheat genotypes at the two developmental stages (15 and 30 DPAs). The figure panels show heat map for traits **(A)** GL, **(B)** GW, **(C)** GLWR, and **(D)** TGW. The genotype names are suffixed with 15 and 30, which indicate number of days after post-anthesis. Red indicates higher gene expression while green represents lower gene expression level; the gene expression levels are log2 transformed.

Expression analysis demonstrated that only 123 genes were expressed in both stages (15 and 30 DPA) of small and large seeded genotypes, of which 23, 33, 27, and 28 were uniquely associated with GL, GW, GLWR, and TGW respectively. Among the identified genes, those with foldchange ≥1 and p-value<0.05 were considered as significantly differentially expressed genes. A total of 18 and 12 genes were significantly differentially regulated in large seeded cultivars at 15 and 30 DPA respectively. At 15 DPA, 11 genes were upregulated and 7 genes were downregulated, while 6 genes were significantly upregulated and downregulated in large seed cultivars at 30 DPA (**Figure 8**). Many genes, including *TraesCS7B02G462900* (SNP-AX-94472687; *Q.GW-7B*), *TraesCS2D02G132600* (SNP-AX-94499721; *Q.GLWR-2D*), *TraesCS1A02G187000* (SNP-AX-95120969; *Q.TGW-GW-1A*), *TraesCS2B02G260200* (SNP-AX-95129853; *Q.GLWR-GL-2B*), and *TraesCS6A02G379200* (SNP-AX-95151036; *Q.GW-6A*), were downregulated at both the time points while *TraesCS1A02G427400* (SNP-AX-94605845; *Q.TGW-1A*), *TraesCS3D02G002700* (SNP-AX-94642652; *Q.GW-3D*), *TraesCS7A02G111200* (SNP-AX-94820170; *Q.GLWR-GL-7A*), and *TraesCS3A02G180200* (SNP-AX-94960788; *Q.GL-3A*) genes were upregulated at 15 as well as 30 DPA. Result also showed that *Q.GLWR-5B* (SNP-AX-94915493*)* associated with GLWR was located within gene *TraesCS5B02G552400* (hypothetical protein), which was only expressed in small grain cultivars with 10 fold upregulation, which showed it has some specific role in small seed cultivars. Another gene, namely *TraesCS7D02G463100*, located within the QTN*Q.GW-7D* and identified in E_1_ and E_5_ location was upregulated in large grain cultivars. *TraesCS7D02G463100* is nuclear transcription factor associated with GLWR.

## Discussion

Grain yield is a highly complex agronomic trait, governed by several genes and also influenced by environmental conditions (Li et al., 2022). It is essentially determined by two main components i.e., number of grains per m^2^ and thousand grain weight (TGW) (Sun et al., 2009; Kumari et al., 2018; Li et al., 2019). In the breeding history, grain yield was mainly improved with increase in the grain number per m^2^, which is determined by grain number per spike (Kumari et al., 2018). In the present study, we focused on the grain shape traits i.e. GL, GW, and GLWR, which determine TGW, a phenotypically stable yield contributing trait and used by the breeders for selecting high yielding varieties (Avni et al., 2018; Duan et al., 2020). The TGW and other grain size traits contribute to higher grain yield than grain number per spike. (Ji et al., 2022). Thus, it is very important to study the grain shape traits when the aim is to improve grain yield. Here, we have applied GWAS to identify genomic regions regulating variation for grain yield in a sub-set of the Indian National Genebank wheat mini core set germplasm (125 accessions). These mini core set accessions have been identified from a core set (2226 accessions), constituted from the entire wheat accessions (22416) conserved in the National Genebank of India (Phogat et al., 2020). Therefore, the mini core set accessions are a valuable genetic resource for mapping various desirable traits including grain shape traits.

The phenotyping of wheat mini core set accessions across the seven environments revealed significant variability among wheat accessions for grain parameters. High coefficients of variation for TGW under all the environments indicated broad phenotypic variation and a large improvement potential. Heritability is the proportion of genotypic variance to all observable variance in the total population. Over the environments, heritability was high for GL and moderate for GW, GLWR, and TGW. The trend of heritability is more specific to environment than traits, as we observed low/moderate heritability for E_5_ and E_6_. These environments fall in stress prone areas affected by less rainfall and high temperatures, which might have caused low heritability of the traits. Promising accessions for grain parameters were identified. Among them, EC578134, IC539313, IC535217, EC464070, and EC578152 were promising for GL as well as TGW. EC339611, EC578134, and IC535217 were promising for GL as well as GLWR, whereas IC335715 was promising for GW and TGW. These accessions can be used in breeding programs for trait introgression, genetics, and genomics study. The significant positive correlation of grain length and width with thousand grain weight revealed that the selection of grains with increased width and length can greatly contribute to grain weight and indirectly to grain yield. Earlier studies have reported moderate to strong correlations between TGW and size (Rasheed et al., 2018). Simmonds et al. (2016) reported that GL and GW in tetraploid and hexaploid wheat can greatly influence the TGW, as longer and broader grains have more starch accumulation and, hence, a higher weight (Simmonds et al., 2016). Previous studies have also reported positive associations among TGW, GL, and GW (Breseghello and Sorrells, 2007; Ramya et al., 2010). Principal component analysis also found GW, TGW, and GL as major contributing traits positively contributing to variations in grain shape among wheat genotypes (**Figure 1**). In our study, the association between GW and GLWR is consistent and significantly negative in all environments. A negative correlation between GW and GLWR could be attributed to compensation of photosynthates to GW rather than to GL. The different correlations could be explained by the influence of the environment on the plant growth and grain development. This study shows that GL, GW, and GLWR are all expected to increase with TGW, one of the major yield components of grain yield and which can be targeted to enhance wheat yield potential. Genetic diversity and population structure in the wheat mini core subset was analyzed using 35K wheat SNP array. Both STRUCTURE and PCA analyses revealed two subpopulations in the wheat mini core set germplasm used in our study. The whole genome LD decay distance in the wheat mini core set was 1.93Mb. Further, LD decay was most rapid in A genome followed by D and B sub-genome. Many earlier studies in wheat have reported much longer LD decay distance ranging from 4Mb to 15 Mb or even more (Pang et al., 2020; Hanif et al., 2021; Li et al., 2021). This suggested the presence of a big LD block size, which has so far limited high-resolution trait mapping in wheat. One of the ways to overcome this problem is to use a very high density genic-SNP array having lakhs of SNPs derived from the coding regions for genotyping of association panels used for conducting GWAS. The high-density genotyping would facilitate construction of haplotypes maps of the associated regions that may help us in pinpointing the exact causal SNP/genes for the target traits.

The Genome-wide association study (GWAS) has been found to be a powerful tool to investigate genetic bases of complex traits in many plant species such as rice, maize, soybean, and wheat (Zegeye et al., 2014; Zhang et al., 2015; Spindel et al., 2016; Zhang et al., 2018; Chaurasia et al., 2020; Chaurasia et al., 2021). There are many statistical methods based on different algorithms that can be used to predict the true association between SNP markers and corresponding phenotypic variations in GWAS (Spindel et al., 2016). In our study, we used the ML-GWAS method for the detection of marker trait-associations for grain shape traits. Multi-locus methods are effective because of their higher statistical power which provides higher efficiency and accuracy for QTNs detection. In numerous studies, it has been found that ML-GWAS is much better than other methods (Bennett et al., 2012; Visioni et al., 2013; Spindel et al., 2016; Ma et al., 2018; Xu et al., 2018; Khan et al., 2019). Peng et al. (2018) used six ML-GWAS models to detect the genetic dissection of 20 free amino acid (AA) levels in *T. aestivum* and claimed that ML-GWAS methods are more reliable and powerful. In the current study, we used five multi-locus methods, mrMLM, FASTmrMLM, FASTmrEMMA, pLARmEB, and ISIS EM-BLASSO, to perform GWAS analysis of four agronomic traits in our association panel. Among these five models, pLARmB identified the highest number of QTNs (211 SNPs), followed by FASTmrMLM (202), ISI EMBLASSO (177), mrMLM(132), and FASTmrEMMA(62).

### QTNs for thousand grain weight, grain length, grain width and grain length width ratio

QTL for grain yield component traits have been extensively studied and reported on all the 21 chromosomes of wheat (Brinton et al., 2017; Cao et al., 2019; Ma et al., 2019; Ji et al., 2022). In our analysis, a total of 160 reliable QTNs were detected for four grain shape-related traits across the seven locations (**Table 2**; **Supplementary Table 6**).

For grain length, 27 QTNs were detected, which were distributed on 17 wheat chromosomes (chr1B, chr1D, chr2B, chr2D, chr3A, chr3B, chr3D, chr4A, chr4B, chr5A, chr5B, chr5D, chr6B, chr6D, chr7A, chr7B, and chr7D). Among these 27 QTNs, 10 QTNs were major (R^2^ ≥ 10% at least in one GWAS method), of which *Q.GL-4A* (SNP-AX-94839917), *Q.GL-7D* (SNP-AX-94872194), *Q.GL-7A* (SNP-AX-94760450) and *Q.GL-6D* (SNP-AX-94647721) were strongest because their R^2^ value were ≥20%. The *Q.GL-4A* on the chr4A with highest R^2 = ^31.94% may explain a significant proportion of the variation for GL in the wheat mini core germplasm. Moreover, this QTN was identified simultaneously in two environments i.e., E_1_ and E_2,_ and with four different models. The *Q.GL-7D* is located within a gene encoding Thioredoxin M type protein with R^2^ ranging between 4.82% to 21.88%. This QTN was predicted in three different environments i.e., E_1_, E_3,_ and E_4,_ and using three different models, suggesting this could be a reliable QTN contributing to GL variation in wheat. In an earlier study, Thioredoxin has been shown to play an important role in preventing sprouting of developing grains in cereals (wheat and barley) by reducing the intramolecular disulfide bonds of storage proteins and other proteins in the starchy endosperm, and thereby affecting grain yield (Guo et al., 2013).

For grain width, 36 QTNs were identified that were distributed on 17 wheat chromosomes. Among these QTNs*, Q.GW-3D* (SNP-AX-94642652), *Q.GW-5B* (SNP-AX-94547840), *Q.GW-3A* (SNP-AX-94741529)*, Q.GW-4D *(SNP-AX-95213549), *and Q.GW-2B* (SNP-AX-94519462 ) were predicted as major QTNs as the phenotypic variance explained by these QTLs was ≥10% of at least one of the ML- GWAS models. *Q.GW-3D and Q.GW-5B* were annotated as unnamed protein product and hypothetical protein respectively. Additionally, *Q.GW-2B* and *Q.GW-4D* had R^2^ values ranging from 7.7 to 12.23 and 0.72 to 17.72, respectively. *Q.GW-2B* was identified at two environments E_3_ and E_4,_ while *Q.GW-4D* was identified at E_2_. Interestingly, both intragenic SNPs showed higher expression in large grain wheat cultivars than small seed cultivars. This suggested that these QTNs might have important roles in determining variation for GW in wheat.

Thirty-seven and thirty-five QTNs were predictive for GLWR and TGW traits respectively. The GLWR-associated QTNs were distributed on 17 chromosomes (chr1A, chr1D, chr2A, chr2B, chr2D, chr3A, chr3B, chr3D, chr4A, chr4B, chr5A, chr5B, chr6A, chr6B, chr6D, chr7B, and chr7D) while QTNs for TGW were spread over 16 chromosomes (chr1A, chr1B, chr1D, chr2A, chr2B, chr2D, chr3A, chr3B, chr3D, chr4B, chr4D, chr5A, chr5D, chr6A, chr7B, and chr7D). In TGW, a total of fourteen SNPs had R^2^≥10 and were considered as major genomic regions for this trait. *Q.TGW-1A* (SNP-AX-94605845) was annotated as TTL1 protein (TETRATRICOPEPTIDE-REPEAT THIOREDOXIN-LIKE 1) with R^2 = ^11.78% and highly expressed in large grains. Studies have reported that *TTL1* positively regulates the stress response regulated by ABA (Guo et al., 2013). The loss of *TTL1* function causes plants to be sensitive to salt and osmotic stress during seed germination and later development (Rosado et al., 2006). So, it could be possible that the identified genomic region in our study may positively regulate the expression of *TTL1* gene and regulate seed maturation. *Q.TGW-5D* (SNP-AX-95234313) was located within a gene encoding cytochrome 450 and it was only identified at E4 environment with R^2 = ^21.45. In a previous study on cytochrome family protein, CYP78A3 on chr7 has been shown to play an important role in wheat seed development by promoting integument cell proliferation (Ma et al., 2015). Thus, it could be suggested that *Q.TGW-5D* (cytochrome 450) identified in our study might also have some role in seed development. A total of 13 QTNs were associated with GLWR and were considered as strong QTNs explaining ≥10% phenotyping variance of the trait. Most of the QTNs were annotated to be either hypothetical proteins or intergenic SNPs. Three QTNs for TGW, namely *Q.GLWR-2D* (SNP-AX-94922377), *Q.GLWR-1A* (SNP-AX-95213485), and *Q.GLWR-6A* (SNP-AX-94722285), were simultaneously identified in three different environments and located on chr2D, chr1A, and chr6A respectively.

### Comparison of the QTLs identified in the present and previous studies

In wheat, several candidate genes underlying grain size and weight have been identified including *TaGS* (Bernard et al., 2008), *TaGW2* (Su et al., 2011), *TaGS-D1* (Zhang et al., 2014), *TaCWI* (Jiang et al., 2015), and *Tackx4* (Chang et al., 2015). Additionally, McCartney et al. (2005) identified two major QTLs for TKW responsible for reduced plant height that were near the *Rht-B1b* and *Rht-D1b* genes that control plant height (McCartney et al., 2005; Gao et al., 2015). Another QTL, *Qtgw-cb.5A*, was identified as a key determinant of final grain weight which increased grain length by driving pericarp cell expansion (Brinton et al., 2017). We performed the comparative analysis of QTNs for grain shape identified in the present study with previously reported QTLs on the basis of their physical locations on chromosomes. Some of the previously reported grain size-associated QTLs were also predicted in our analysis. For example, *Qgl.cib-CK1-4A* associated with GL on chr4A coincided with *Q.GL-4A* (SNP-AX-94839917) for grain length trait at the same region on chr4A and identified in two environments (E_1_ and E_2_). Further, LOD (4.92~6.13) and R2 value (15.33~31.94) of this QTL demonstrated its importance in regulating GL trait. Goel et al. (2019) identified *qTKW.6A.1* associated with TGW on 6A at the interval 166.64-596.18 Mb (Goel et al., 2019). The QTN, *Q.TGW-6A* (SNP-AX-95240001), identified in our study appears to correspond to *qTKW.6A.1.* Interestingly, *Q.TGW-6A* was identified at two locations, E2 and E4, which showed that it is a stable genomic region for TGW. Further, we found that *Q.GL-TGW-6A* (SNP-AX-95238912) and *Q.GLWR-6A* (SNP-AX-94722285), which are located on chr6A at 362.7Mb and 307Mb, overlapped with the grain shape QTLs identified by Cao et al. (2019) and Ji et al. (2022), respectively. Interestingly, *Q.GL-TGW-6A* was identified in three environments (E3, E4, and E5). LOD score and R^2^ value of *Q.GL-TGW-6A and Q.GLWR-6A* ranged from 3.46 to 8.23 and 2.6 to 23.8 respectively. On the other hand, *Q.GLWR-6A* was present at 307Mb on chr6A with LOD score (3.47~7.59) and R2 value (5.03~18.87). Since the two QTNs on chr6A were also identified in the previous studies, these appear to represent major genomic regions for the grain shape traits.

A few other underlying genes influenced grain size and weight have been reported by Cabral et al., 2018. *TaGS-D1*, controlling GL and grain weight, is an ortholog of *OsGs3 and* located at 106.73 Mb on chr3D. Expression pattern of this *TaGS-D1* (*TraesCS7A03G0037700*) gene in our data showed relatively higher expression in large seeded genotypes as compare to small seeded genotypes. So, we examined nearby QTNs around the gene and we found two QTNs, *Q.GLWR-7*D and *Q.TGW-7D*, located in the vicinity of *TaGS-D1* and positioned at 54.9Mb on chr7DS and 100.1 Mb on chr7D respectively. The presence of these two QTNs indirectly suggested a major locus which corresponds to either *TaGS-D1* or an additional novel gene for grain shape trait on the short of chr7D. A second grain weight locus cytokinin oxidase/dehydrogenase (*TaCKX6-D1*) gene is physically located on chromosome 3D and played a key role in controlling cytokinin levels and affects grain weight in wheat (Zhang et al., 2012). *TaCKX6-D1* gene is located at 106.73 Mb on chr3D, so its expression could be influenced by nearby SNPs around the gene. On the basis of the physical location of gene, we found two significant genes, *Q.GL-GW-3D* and *Q.TGW-3D* (SNP-AX-95008504, and SNP-AX-94406908), in our analysis at 151.4 Mb and 239.3 Mb respectively. *Q.GL-GW-3D* associated with GL was identified in four environments (E_1_, E_2_, E_4_, and E_5_) with LOD value from 3.05 to 6.33 and R^2^ value from 4.09 to 14.97, which showed the significance of SNP. The second, *Q.TGW-3D*, demonstrated association with TGW with LOD (3.16~8.2) and R^2^ (7.21~11.48) and was identified at E2, E3, and E4 environments. Both the QTNs were annotated as hypothetical proteins and expressed in our transcriptome data. *Q.TGW-3D* was highly expressed in large seed cultivars while *Q.GL-GW-3D* also showed expression in both cultivars. In conclusion, in this study we have comprehensively phenotyped wheat mini core germplasm accessions for grain shape traits and identified promising accessions for large grain size and length which can be incorporated in breeding programs. Further, integration of phenotyping and genotyping data has enabled us to identify genomic regions/candidate genes, some of which are novel. Comparative study also showed that many QTNs identified in our study represented novel genomic regions that can be further validated for their role in determining grain size and can be potentially exploited in breeding programs to develop high-yielding varieties.

## Data availability statement

The datasets presented in this study can be found in online repositories. The names of the repository/repositories and accession number(s) can be found below: https://www.ncbi.nlm.nih.gov/, PRJNA906296.

## Author contributions

JK planned and handled the field experiments, conducted formal analysis, and assisted in writing the manuscript. DL contributed to the data curation, investigation, formal analysis, and original draft writing. MY and ST planned and executed DNA extraction and SNP data generation. PJ, SS, ST, SM, and HA performed data analysis, writing, reviewing, and editing, NS, KS and KM recorded phenotyping data and reviewed and edited the manuscript, RS, MY, and GS contributed resources and participated in the writing, reviewing, and editing. AS contributed to the conceptualization, supervision, writing, reviewing, and editing of the manuscript. All authors contributed to the article and approved the submitted version.
