# Supplementary material for: Association mapping reveals novel genes and genomic regions controlling grain size architecture in mini core accessions of Indian National Genebank wheat germplasm collection
Source: Front Plant Sci. 2023 Jun 28;14:1148658. doi: 10.3389/fpls.2023.1148658 (PMC10345843; doi:10.3389/fpls.2023.1148658)
Supplement: Supplementary file 1 [file DataSheet_1.pdf]

DL-Y1

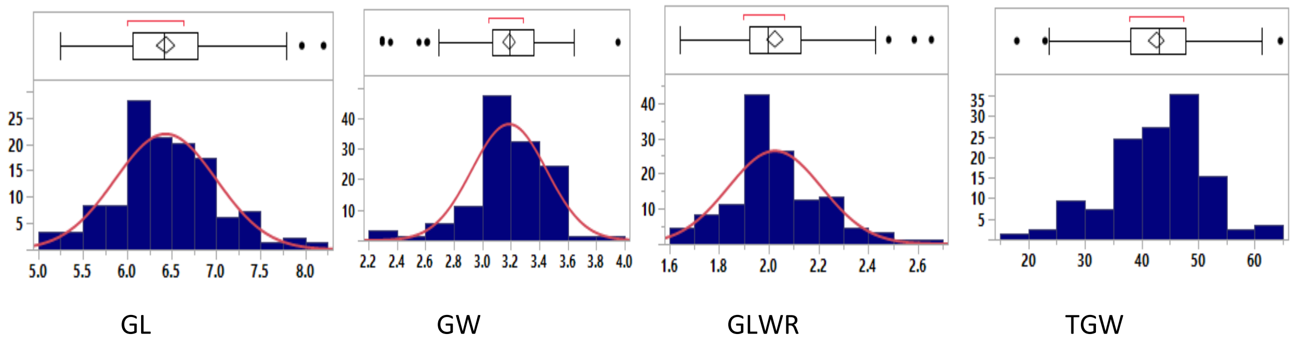

DL-Y2

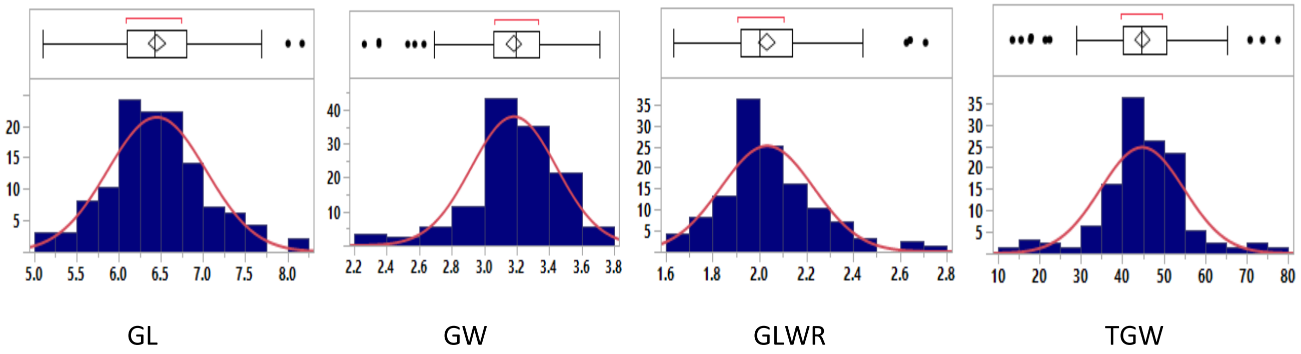

DL-Y3

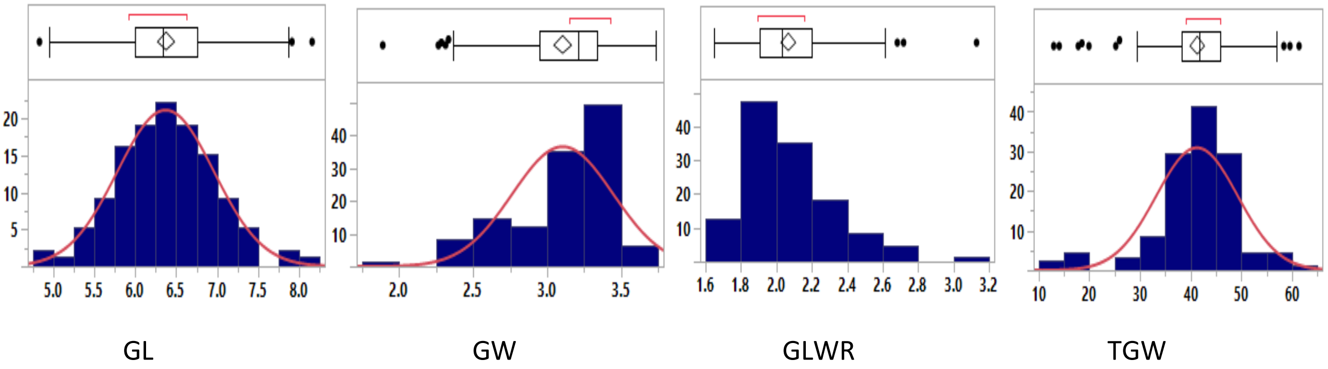

DL-Y4

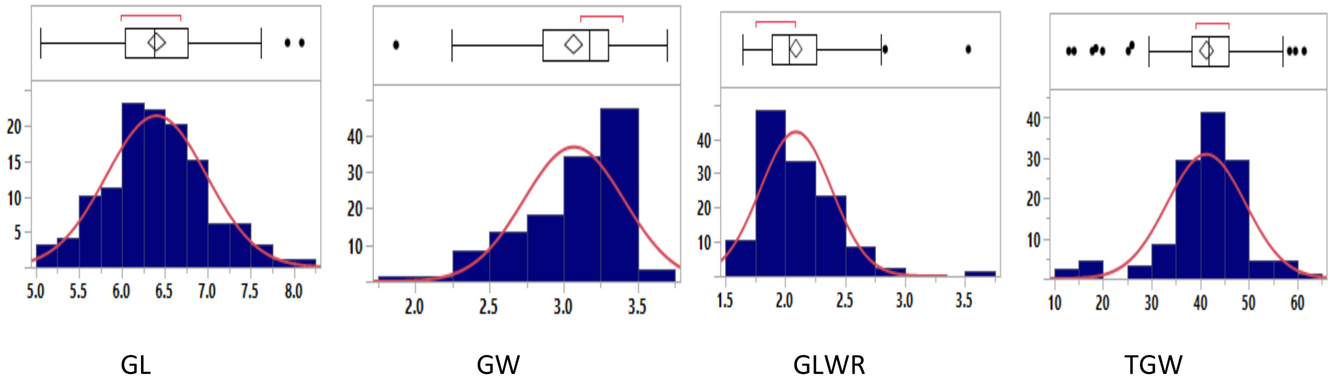

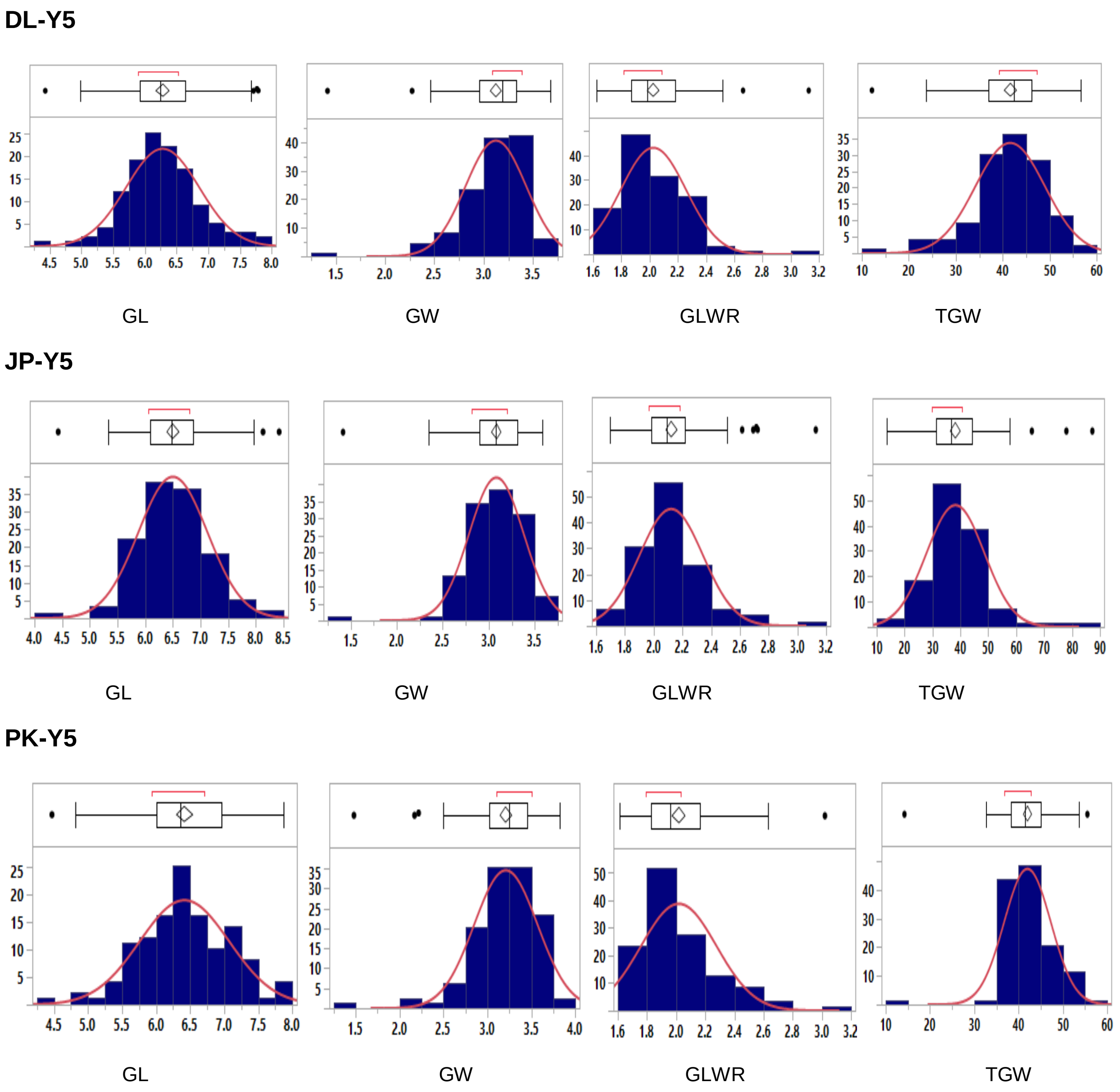

Supplementary Figure S1. Histogram depicting variation in grain parameters under seven environments. Y-axis represent the number of accessions and X-axis represent the grain quality traits.

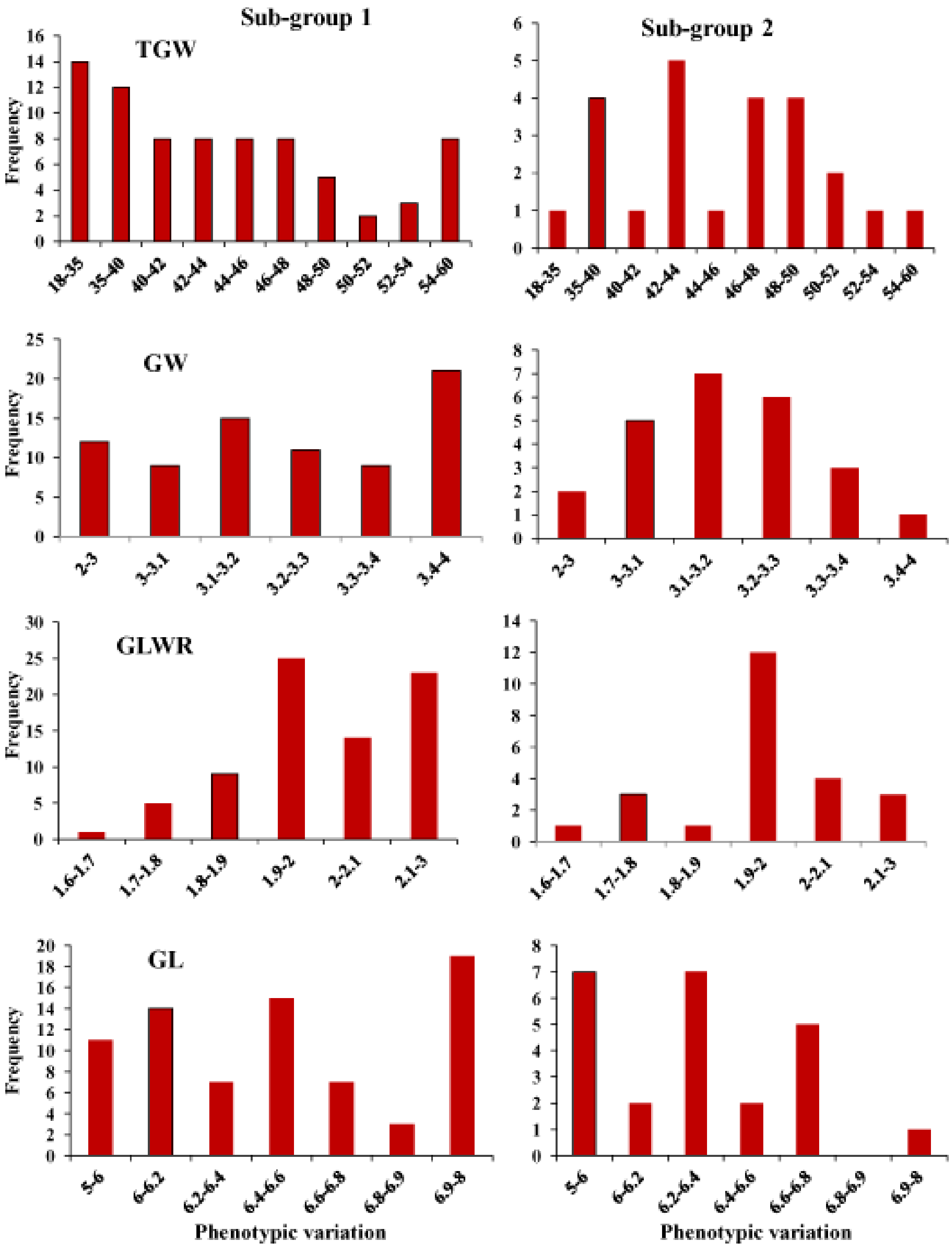

Supplementary Figure S2. Histogram depicting phenotypic variation among the members of the same gene pool.

Supplementary Tables

Supplementary Table S1. Details of wheat germplasm used in the study.

S.No	Accession	Collector No/Other ID/Cultivar Name/Pedigree	Collection Source/ Developer Institution /Country
**1**	EC0187159	Germplasm	Israel
**2**	EC021058	Germplasm	ICAR-NBPGR, Delhi, India
**3**	EC0217835	32039	UNITED STATES OF AMERICA
**4**	EC0299335	ICNO-5266	Syrian Arab Republic
**5**	EC0313710	WIR-55181, Brigantina	Ukraine
**6**	EC0313713	WIR-58801, Spartanka	USSR (Russia)
**7**	EC0339611	PI-519264 IPPO 16	UNITED STATES OF AMERICA
**8**	EC0339632	PI-520594	USA
**9**	EC0426644	Tincurrin	Australia
**10**	EC0463396	Germplasm	CIMMYT, Mexico
**11**	EC0464070	CN-98	CIMMYT, Mexico
**12**	EC0528129	NA	NA
**13**	EC0541159	Bruehl	USA
**14**	EC0542279	Germplasm	USA
**15**	EC0573814A	5613	UNKNOWN
**16**	EC0573974	5869	ICAR-NBPGR, Delhi, India
**17**	EC0574037	5952	ICAR-NBPGR, Delhi, India
**18**	EC0574914	9084, Germplasm	ICAR-NBPGR, Delhi, India
**19**	EC0575981	219, Germplasm	ICAR-NBPGR, Delhi, India
**20**	EC0576159	E-10856	USA
**21**	EC0576578	E-320	Australia
**22**	EC0576591	E-3248	ICAR-NBPGR, Delhi, India
**23**	EC0576792	E-514	China
**24**	EC0576816	E-563	ICAR-NBPGR, Delhi, India
**25**	EC0576889	E-7233	USA
**26**	EC0576930	E-7726	USA
**27**	EC0576941	E-7777	ICAR-NBPGR, Delhi, India
**28**	EC0577050	E-931	ICAR-NBPGR, Delhi, India
**29**	EC0577738	E-3288	Finland
**30**	EC0577954	E-3672	ICAR-NBPGR, Delhi, India
**31**	EC0578134	SYNT-E- 33	ICAR-NBPGR, Delhi, India
**32**	EC0578152	SYNT-E- 51	ICAR-NBPGR, Delhi, India
**33**	EC0578153	SYNT-E- 52	ICAR-NBPGR, Delhi, India
**34**	EC0578185	SYNT-E- 85	ICAR-NBPGR, Delhi, India
**35**	EC0609338	07A 37	USA
**36**	EC06903	PI-59183	USA
**37**	EC0697725	NA	NA

**38**	IC0111787	RAJ-164	Rajasthan, India
**39**	IC0116274	Ajanta (BDN 519)	Maharashtra, India
**40**	IC0116276	Ajanta (BDN-519)	Maharashtra, India
**41**	IC0122726	NA	NA
**42**	IC0128150	BW-11	West Bengal, India
**43**	IC0128151	C-306	IIWBR, Karnal, Haryana, India
**44**	IC0128280	HPW-283	Rajasthan, India
**45**	IC0145237	BANSI-224	IIWBR, Karnal, Haryana, India
**46**	IC0145522	NA	NA
**47**	IC0252419	BW/SH-9	West Bengal, India
**48**	IC0252422	BW/SH-14	West Bengal, India
**49**	IC0252429	BW/SH-28	West Bengal, India
**50**	IC0252440	BW/SH-42	West Bengal, India
**51**	IC0252469	C-578	West Bengal, India
**52**	IC0252668	HP-1704	Bihar, India
**53**	IC0252699	HS-361	ICAR-NBPGR, Delhi, India
**54**	IC0252772	J-496	ICAR-NBPGR, Delhi, India
**55**	IC0252794	K-9351	CSUA&T, Kanpur,Uttar Pradesh, India
**56**	IC0252796A	K-9362	CSUA&T, Kanpur,Uttar Pradesh, India
**57**	IC0252928	RW-482	ICAR-NBPGR, Delhi, India
**58**	IC0252954	UP-2425 (HD-230/UP-2263)	Uttar Pradesh, India
**59**	IC026720	NA	NA
**60**	IC026728	Germplasm	Himachal Pradesh, India
**61**	IC028029	Germplasm	ICAR-NBPGR, Delhi, India
**62**	IC0282300	Halana	Uttar Pradesh, India
**63**	IC028584	Germplasm	Gujarat, India
**64**	IC028622	CN-47	Gujarat, India
**65**	IC028649	CN-218	Gujarat, India
**66**	IC028755	A-206	Gujarat, India
**67**	IC028872	CN-72	Rajasthan, India
**68**	IC028889	CN-92, Germplasm	Rajasthan, India
**69**	IC029002	Raj-234	Rajasthan, India
**70**	IC029008	Raj-240	Rajasthan, India
**71**	IC0290161	NA	NA
**72**	IC0290186	Germplasm	ICAR-NBPGR, Delhi, India
**73**	IC0290195	Germplasm	ICAR-NBPGR, Delhi, India
**74**	IC0290234	KRL-19	IIWBR, Karnal, Haryana, India
**75**	IC0303067	HW-2045 (Kaushambi),(HD2402*6//SUNSTAR*6/C-80-1)	IARI, New Delhi, India
**76**	IC0309875	PBW-373	PAU, Ludhiana, Punjab, India
**77**	IC0335540	Kharachia-65	IIWBR, Karnal, Haryana, India
**78**	IC0335683	EGPSN(3)-71	IIWBR, Karnal, Haryana, India
**79**	IC0335715	EGPYT(IST)-24	ICAR-NBPGR, Delhi, India
**80**	IC0335776	CPAN 6211	ICAR-NBPGR, Delhi, India
**81**	IC0335998	UDND/01-166//	Karnataka

**82**	IC035163	Tadia-Genepool	IIWBR, Karnal, Haryana, India
**83**	IC0401927	GW-18	IIWBR, Karnal, Haryana, India
**84**	IC0402042	K-9006	IIWBR, Karnal, Haryana, India
**85**	IC0406521	AKS/RRA/BS-171	Uttarakhand, India
**86**	IC0406688	KCM-669	Uttarakhand, India
**87**	IC0443633	GW-1170	IIWBR, Karnal, Haryana, India
**88**	IC0443766	RAJ-3765 (HD-2402/VL-639)	Dungarpur, Rajasthan, India
**89**	IC0449061	K-9423/Unnat Halna	CSUA&T, Kanpur,Uttar Pradesh, India
**90**	IC0527448	HI-1531	Madhya Pradesh, India
**91**	IC0529051	VWFW-1247	Uttarakhand, India
**92**	IC0530089	VWFW-994	Uttarakhand, India
**93**	IC0530131	VWFW- 1424	Uttarakhand, India
**94**	IC0531012	WL-3299	PAU, Ludhiana, Punjab, India
**95**	IC0531062	WL 5630	PAU, Ludhiana, Punjab, India
**96**	IC0531183	WG- 3285	PAU, Ludhiana, Punjab, India
**97**	IC0531524	W 6496	PAU, Ludhiana, Punjab, India
**98**	IC0532019	Kohinoor-83	Himachal Pradesh, India
**99**	IC0533610	PI-430043	ICAR-NBPGR, Delhi, India
**100**	IC0534929	PI-348983	ICAR-NBPGR, Delhi, India
**101**	IC0534949	PI-383307	ICAR-NBPGR, Delhi, India
**102**	IC0535217	NC-55612	ICAR-NBPGR, Delhi, India
**103**	IC0535330	NC-57989	ICAR-NBPGR, Delhi, India
**104**	IC0539313	Tadia-Genepool	IIWBR, Karnal, Haryana, India
**105**	IC0539314	TADIA-1	IIWBR, Karnal, Haryana, India
**106**	IC0539317	TADIA-4	IIWBR, Karnal, Haryana, India
**107**	IC0542051	MIYCSN-22	ICAR-NBPGR, Delhi, India
**108**	IC0542063	MFSYCINT-166	ICAR-NBPGR, Delhi, India
**109**	IC0542076	5-IAT-32	ICAR-NBPGR, Delhi, India
**110**	IC0543373	IDTN-70 (ET-90550)	ICAR-NBPGR, Delhi, India
**111**	IC0547649	9th-EGPSN-11	ICAR-NBPGR, Delhi, India
**112**	IC0554661	WH-1021	IIWBR, Karnal, Haryana, India
**113**	IC0566636	PHR-1011	IIWBR, Karnal, Haryana, India
**114**	IC0574387	HD 2985	Division Of Genetics IARI Delhi Delhi
**115**	IC0574388	HD -2987	ICAR-NBPGR, Delhi, India
**116**	IC0574476	HD-2967	ICAR-NBPGR, Delhi, India
**117**	IC0576640	NA	NA
**118**	IC0582717	HPW-283	Himachal Pradesh, India
**119**	IC075221	NA	NA
**120**	IC075240	NA	NA
**121**	IC0822335A	NA	NA
**122**	IC082425A	Germplasm	ICAR-NBPGR, Delhi, India
**123**	Kharchia65	NA	NA
**124**	Ningmai	NA	NA
**125**	Chunmai	NA	NA

Supplementary Table S2. Descriptive statistics for grain yield related traits under seven environments.GL, grain length; GW, grain weight; GLWR, grain length and width ratio; TGW, thousand grain weight * E1, Delhi (2015-16); E2, Delhi (2016-17); E3, Delhi (2017-18); E4, Delhi (2018-19); E5, Delhi (2019-20); E6, Powarkheda (2019-20); E7, Jodhpur (2019–20).

Traits	Mean	Std Dev	Coefficient of Varaition	Minimum	Maximum
			**E1**		
**GL**	6.4	0.6	8.9	5.2	8.2
**GW**	3.2	0.3	8.4	2.3	4
**GLWR**	2	0.2	9.8	1.5	2.7
**TGW**	42.5	8.2	19.5	18	64.5
			**E2**		
**GL**	6.4	0.6	9.2	5.1	8.2
**GW**	3.2	0.3	8.3	2.3	3.7
**GLWR**	2	0.2	10.5	1.3	2.7
**TGW**	45	9.2	20.4	13.5	67.6
			**E3**		
**GL**	6.4	0.6	9.4	4.8	8.2
**GW**	3.1	0.3	11.1	1.9	3.7
**GLWR**	2.1	0.2	11.9	1.6	3.1
**TGW**	37.5	7.4	19.7	7.9	51.9
			**E4**		
**GL**	6.4	0.6	9.1	5	8.1
**GW**	3.1	0.3	11.1	1.9	3.7
**GLWR**	2.1	0.3	14.1	1.6	3.5
**TGW**	40.7	8.8	21.6	9.9	61.4
			**E5**		
**GL**	6.3	0.6	9.2	4.4	7.8
**GW**	3.1	0.3	9.9	1.4	3.7
**GLWR**	2	0.2	11.5	1.6	3.1
**TGW**	41.6	7.4	17.8	12.2	56.7
			**E6**		
**GL**	6.5	0.6	9.6	4.4	8.4
**GW**	3.1	0.3	9.7	1.4	3.6
**GLWR**	2.1	0.2	10.4	1.7	3.1
**TGW**	38.1	9.4	24	13.8	67.2
			**E7**		
**GL**	6.4	0.7	10.3	4.5	7.9
**GW**	3.2	0.4	11.3	1.5	3.8
**GLWR**	2	0.3	12.9	1.6	3
**TGW**	41.9	5.3	12.6	14.3	55.5

Supplementary Table S3. Detail statistics for grain yield traits parameters under different environments.

Environment	Statistic	BLUP_GL	BLUP_GW	BLUP_GLWR	BLUP_TGW
**E1**	Heritability Gen	0.632	0.280	0.220	0.249
**E1**	Genotype Variance	0.205	0.086	0.116	17.879
**E1**	Residual Variance	0.120	0.098	0.047	53.874
**E1**	Grand Mean Gen	6.337	3.196	2.020	42.609
**E1**	Avg Std Err Difference Gen	0.403	0.060	0.020	5.244
**E1**	LSD Gen	0.797	0.120	0.040	10.372
**E2**	Heritability Gen	0.710	0.242	0.460	0.284
**E2**	Genotype Variance	0.242	0.038	0.044	30.805
**E2**	Residual Variance	0.099	0.120	0.051	77.630
**E2**	Grand Mean Gen	6.343	3.199	2.025	44.309
**E2**	Avg Std Err Difference Gen	0.393	0.070	0.020	6.736
**E2**	LSD Gen	0.777	0.140	0.040	13.323
**E3**	Heritability Gen	0.779	0.374	0.477	0.741
**E3**	Genotype Variance	0.265	0.070	0.063	0.770
**E3**	Residual Variance	0.075	0.117	0.069	61.996
**E3**	Grand Mean Gen	6.285	3.105	2.068	37.660
**E3**	Avg Std Err Difference Gen	0.364	0.188	0.151	3.001
**E3**	LSD Gen	0.720	0.371	0.298	5.910
**E4**	Heritability Gen	0.824	0.602	0.415	0.270
**E4**	Genotype Variance	1.170	0.191	0.202	31.772
**E4**	Residual Variance	0.097	0.127	0.092	85.872
**E4**	Grand Mean Gen	6.230	3.020	2.080	39.857
**E4**	Avg Std Err Difference Gen	0.448	0.397	0.169	6.844
**E4**	LSD Gen	0.886	0.785	0.334	13.530
**E5**	Heritability Gen	0.786	0.576	0.623	0.666
**E5**	Genotype Variance	0.256	0.055	0.032	37.044
**E5**	Residual Variance	0.070	0.041	0.019	18.613
**E5**	Grand Mean Gen	6.272	3.221	1.951	43.074
**E5**	Avg Std Err Difference Gen	0.342	0.220	0.158	5.084
**E5**	LSD Gen	0.676	0.435	0.312	10.052
**E6**	Heritability Gen	0.325	0.288	0.332	0.218
**E6**	Genotype Variance	0.047	0.000	0.016	16.570
**E6**	Residual Variance	0.329	0.083	0.032	88.592
**E6**	Grand Mean Gen	6.523	3.083	2.125	38.358
**E6**	Avg Std Err Difference Gen	0.287	0.414	0.147	5.305
**E6**	LSD Gen	0.568	0.903	0.290	10.489
**E7**	Heritability Gen	0.225	0.223	0.259	0.226
**E7**	Genotype Variance	0.062	0.025	0.015	6.230
**E7**	Residual Variance	0.436	0.141	0.079	21.319
**E7**	Grand Mean Gen	6.644	3.209	2.131	42.006

E7	Avg Std Err Difference Gen	0.630	0.414	0.187	3.124
E7	LSD Gen	1.360	0.902	0.405	6.178

Supplementary Table S4. Description of phenotypic correlations between grain yield parameters in seven environments.

Environments/Traits				
**E1**				
	**GL**	**GW**	**GLWR**	**TGW**
**GL**	1.000			
**GW**	0.3682**	1.000		
**GLWR**	0.5852**	-0.363**	1.000	
**TGW**	0.4719**	0.371**	0.055	1.000
**E2**				
**GL**	1.000			
**GW**	0.406**	1.000		
**GLWR**	0.562**	-0.438**	1.000	
**TGW**	0.394**	0.380**	0.015	1.000
**E3**				
**GL**	1.000			
**GW**	0.444**	1.000		
**GLWR**	0.335**	-0.684**	1.000	
**TGW**	0.538**	0.604**	-0.242*	1.000
**E4**				
**GL**	1.000			
**GW**	0.225*	1.000		
**GLWR**	0.457**	-0.748**	1.000	
**TGW**	0.428**	0.488**	-0.189*	1.000
**E5**				
**GL**	1.000			
**GW**	0.406**	1.000		
**GLWR**	0.361**	-0.686**	1.000	
**TGW**	0.643**	0.723**	-0.233*	1.000
**E6**				
**GL**	1.000			
**GW**	0.498**	1.000		
**GLWR**	0.390**	-0.583**	1.000	
**TGW**	0.483**	0.423**	0.010	1.000
**E7**				
**GL**	1.000			
**GW**	0.424**	1.000		
**GLWR**	0.368**	-0.670**	1.000	
**TGW**	0.233*	0.220*	-0.092	1.000

Supplementary Table S5. Description of phenotypic correlations between different environments for traits.

		Phenotypic Correlations					
	**Env**	**E1**	**E2**	**E3**	**E4**	**E5**	**E6**
**GL**	**E2**	0.985					
	**E3**	0.664	0.668				
	**E4**	0.985	1.000	0.668			
	**E5**	0.693	0.695	0.702	0.393		
	**E6**	0.219	0.234	0.245	0.089	0.214	
	**E7**	0.104	0.121	0.084	0.035	0.179	0.643
	**Env**	**E1**	**E2**	**E3**	**E4**	**E5**	**E6**
**GW**	**E2**	0.953					
	**E3**	0.465	0.453				
	**E4**	0.464	0.451	1.000			
	**E5**	0.529	0.518	0.312	0.268		
	**E6**	0.259	0.249	0.131	0.001	0.289	
	**E7**	0.313	0.343	0.241	0.156	0.387	0.463
	**Env**	**E1**	**E2**	**E3**	**E4**	**E5**	**E6**
**GLWR**	**E2**	0.961					
	**E3**	0.522	0.522				
	**E4**	0.526	0.532	0.839			
	**E5**	0.565	0.574	0.506	0.322		
	**E6**	0.278	0.296	0.194	0.089	0.319	
	**E7**	0.139	0.188	0.203	0.117	0.261	0.626
	**Env**	**E1**	**E2**	**E3**	**E4**	**E5**	**E6**
**TGW**	**E2**	0.622					
	**E3**	0.700	0.755				
	**E4**	0.664	0.954	0.859			
	**E5**	0.390	0.496	0.432	0.438		
	**E6**	-0.027	0.023	0.037	0.001	0.144	
	**E7**	0.047	0.104	0.120	0.091	0.121	0.121

Supplementary Table S6. The significant QTNs for four wheat yield-related traits detected simultaneously by using two or more multi-locus GWAS methods and at only one location.1, mrMLM; 2, FASTmrMLM; 3, FASTmrEMMA; 4, pLARmEB; 5, ISIS EM-BLASSO.

QTN	SNP	Trait	Location	Chr	Position	LOD>3	R^2	Models
**Q.GL-1B**	**AX-94631915**	GL	E5	chr1B	5.89E+08	3.09~4.86	2.63~8.06	**1,3,4,5**
**Q.GL-1D**	**AX-94613367**	GL	E4	chr1D	4.14E+08	3.15~3.36	1.95~3.18	**1,2**
**Q.GL-2B**	**AX-94450478**	GL	E4	chr2B	7.31E+08	7.78~8.1	17.82~19.55	**1,2**
**Q.GL-2D**	**AX-94866977**	GL	E4	chr2D	11393132	5.56~8.28	6.07~9.32	**1,2**
**Q.GL-2D**	**AX-95097906**	GL	E3	chr2D	4.67E+08	3.89~14.32	2.68~16.41	**3,4,5**
**Q.GL-3A**	**AX-94960788**	GL	E3	chr3A	2.08E+08	6.41~6.44	2.88~4.66	**4,5,**
**Q.GL-3B**	**AX-94590750**	GL	E3	chr3B	7.24E+08	3.52~4.41	1.96~2.44	**4,5,**
**Q.GL-4B**	**AX-95167555**	GL	E2	chr4B	66811553	3.86~4.51	5.42~12.81	**1,2,**
**Q.GL-5A**	**AX-94410486**	GL	E7	chr5A	5.83E+08	4.01~6.8	4.42~7.27	**4,5**
**Q.GL-5B**	**AX-95226251**	GL	E6	chr5B	3.93E+08	4.31~4.64	4.62~7.84	**1,2,4**
**Q.GL-6B**	**AX-94738045**	GL	E5	chr6B	17703691	3.12~3.52	6.15~9.67	**1,2**
**Q.GL-6D**	**AX-94647721**	GL	E6	chr6D	82092246	4.41~5.89	14.34~26.23	**1,2,4,5**
**Q.GL-7A**	**AX-94760450**	GL	E7	chr7A	5051725	3.72~5.68	13.66~28.14	**1,2,5**
**Q.GLWR-1A**	**AX-94678574**	GLWR	E7	chr1A	61382007	3.57~4.24	5.67~8.52	**3,5**
**Q.GLWR-1D**	**AX-94418502**	GLWR	E6	chr1D	2.05E+08	4.23~4.47	2.87~8.52	**1,2,4**
**Q.GLWR-2A**	**AX-94643237**	GLWR	E7	chr2A	6.93E+08	3~4.34	3.14~7.23	**1,3**
**Q.GLWR-2A**	**AX-94687724**	GLWR	E7	chr2A	7.15E+08	3.17~5.83	2.07~5.45	**2,4**
**Q.GLWR-2B**	**AX-94456096**	GLWR	E3	chr2B	6186595	3.48~4.99	0.3~4.97	**4,5**
**Q.GLWR-2B**	**AX-94542840**	GLWR	E7	chr2B	1.10E+08	3.4~6.44	4.73~21.9	**2,4,5**
**Q.GLWR-2B**	**AX-95167429**	GLWR	E6	chr2B	6.62E+08	3.07~4.16	5.49~22.74	**1,2,4,5**
**Q.GLWR-2D**	**AX-94499721**	GLWR	E3	chr2D	77381475	3.27~5.91	1.32~8.64	**2,3,4,5**
**Q.GLWR-2D**	**AX-94412799**	GLWR	E6	chr2D	3.56E+08	3.05~3.22	1.55~5.99	**1,5**
**Q.GLWR-3A**	**AX-94519760**	GLWR	E1	chr3A	30345562	3.24~5.76	0.42~5.73	**1,2,**
**Q.GLWR-3B**	**AX-94612704**	GLWR	E3	chr3B	27895627	3.24~14.64	3.47~12.04	**1,2,4,5**
**Q.GLWR-3B**	**AX-94581734**	GLWR	E3	chr3B	1.33E+08	5.07~6.5	0.75~2.09	**1,2,**
**Q.GLWR-3B**	**AX-94484138**	GLWR	E7	chr3B	4.57E+08	5.63~5.9	5.23~6.21	**2,4,**
**Q.GLWR-3B**	**AX-94624755**	GLWR	E1	chr3B	8.20E+08	3.29~4.73	0.95~5.65	**1,4,5**
**Q.GLWR-3D**	**AX-94798168**	GLWR	E4	chr3D	2194214	6.04~6.51	19.24~20.3	**4,5**
**Q.GLWR-4A**	**AX-94818960**	GLWR	E3	chr4A	5.42E+08	3.1~8.2	4.72~22.79	**1,3,4,5**
**Q.GLWR-4A**	**AX-94624981**	GLWR	E3	chr4A	6.12E+08	3.47~10	1.14~6.73	**1,4**

**Q.GLWR-4B**	**AX-95255558**	GLWR	E6	chr4B	5.36E+08	3.32~3.34	0~3.14	**2,5**
**Q.GLWR-4B**	**AX-94400582**	GLWR	E7	chr4B	6.72E+08	4.04~8.39	7.12~14.66	**4,5**
**Q.GLWR-5A**	**AX-95112173**	GLWR	E7	chr5A	4.96E+08	3.3~4.78	10.87~10.97	**2,4**
**Q.GLWR-5A**	**AX-94862815**	GLWR	E3	chr5A	6.01E+08	3.46~4.16	0~1.09	**2,4**
**Q.GLWR-6B**	**AX-94410929**	GLWR	E6	chr6B	1.24E+08	4.5~7.62	11.29~22.95	**4,5**
**Q.GLWR-6B**	**AX-94893651**	GLWR	E1	chr6B	5.56E+08	8.2~26.55	0.42~1.67	**2,4**
**Q.GLWR-6D**	**AX-94585341**	GLWR	E3	chr6D	4.36E+08	3.05~5.03	4.68~11.62	**1,2,4,5**
**Q.GLWR-7B**	**AX-95107992**	GLWR	E1	chr7B	14728251	3.69~4.89	0.62~9.89	**2,5**
**Q.GLWR-7D**	**AX-94829311**	GLWR	E1	chr7D	54998894	5.51~5.56	7.03~12.85	**1,5**
**Q.GLWR-7D**	**AX-94705837**	GLWR	E3	chr7D	6.09E+08	4.08~6.18	0~6.64	**4,5**
**Q.GW-1B**	**AX-94856268**	GW	E5	chr1B	5.74E+08	6.14~10.75	5.56~7.51	**1,4**
**Q.GW-2D**	**AX-94461313**	GW	E2	chr2D	65692209	3.08~5.71	3.07~3.99	**1,4**
**Q.GW-2D**	**AX-95181452**	GW	E1	chr2D	83142083	5.33~6.2	0.61~7.58	**1,2**
**Q.GW-2D**	**AX-94669780**	GW	E2	chr2D	5.73E+08	3.36~5.07	0.83~3.13	**1,2,4,5**
**Q.GW-2D**	**AX-94598441**	GW	E1	chr2D	6.34E+08	5.37~5.6	0.48~5.81	**4,5**
**Q.GW-3A**	**AX-94741529**	GW	E5	chr3A	19559964	4.13~5.99	15.09~21.54	**1,4,5**
**Q.GW-3A**	**AX-94387611**	GW	E6	chr3A	5.61E+08	3.43~3.77	3.14~9.09	**1,2,5**
**Q.GW-3D**	**AX-94540502**	GW	E6	chr3D	3.61E+08	3.32~6.26	2.86~4.88	**1,5**
**Q.GW-4D**	**AX-95213549**	GW	E2	chr4D	5.09E+08	4.32~5.56	0.72~17.72	**1,2,4**
**Q.GW-5A**	**AX-94590607**	GW	E2	chr5A	4.69E+08	3.92~3.96	4.93~5.78	**1,2**
**Q.GW-5B**	**AX-94547840**	GW	E6	chr5B	4.83E+08	6.61~6.87	18.88~26.32	**1,5**
**Q.GW-5B**	**AX-95113198**	GW	E1	chr5B	5.88E+08	4.52~5.68	1.43~2.29	**2,4,**
**Q.GW-5B**	**AX-94909164**	GW	E1	chr5B	6.97E+08	3.47~3.64	0.47~1.13	**4,5**
**Q.GW-6B**	**AX-94916571**	GW	E1	chr6B	9003693	3.44~3.77	0~0	**2,4**
**Q.GW-6B**	**AX-94435221**	GW	E5	chr6B	93562245	3.06~3.45	1.47~2.07	**2,5**
**Q.GW-7B**	**AX-95209714**	GW	E2	chr7B	23909521	3.19~3.54	0~3.9	**2,5**
**Q.GW-7B**	**AX-94483298**	GW	E2	chr7B	6.90E+08	5.51~6.95	0.73~7.79	**3,4**
**Q.GW-7B**	**AX-94560694**	GW	E6	chr7B	7.04E+08	3.11~3.65	6.77~8.77	**4,5**
**Q.GW-7B**	**AX-94472687**	GW	E5	chr7B	7.20E+08	4.23~4.28	3.81~7.26	**1,2**
**Q.TGW-1A**	**AX-95219658**	TGW	E1	chr1A	4812661	3.04~5.06	9.94~12.03	**1,2,5**
**Q.TGW-1A**	**AX-94990907**	TGW	E1	chr1A	3.72E+08	9.29~10.51	6.65~9.86	**2,4**
**Q.TGW-1A**	**AX-94605845**	TGW	E7	chr1A	5.82E+08	3.05~3.49	3.02~11.78	**1,5**
**Q.TGW-1B**	**AX-94432371**	TGW	E4	chr1B	3.30E+08	4.76~6.59	6.53~12.11	**1,2,3**
**Q.TGW-1B**	**AX-94440309**	TGW	E4	chr1B	5.53E+08	5.49~5.53	3.94~8.49	**1,2,4**

**Q.TGW-1B**	**AX-94396871**	TGW	E2	chr1B	5.54E+08	3.36~6.48	2.04~6.93	**3,4,5**
**Q.TGW-1B**	**AX-94711748**	TGW	E3	chr1B	6.27E+08	3.05~4.33	2.48~5.32	**1,2**
**Q.TGW-1B**	**AX-94429181**	TGW	E3	chr1B	6.43E+08	3.62~4.41	2.79~12.85	**1,2,4**
**Q.TGW-1D**	**AX-95123147**	TGW	E1	chr1D	4.65E+08	4.42~11.74	6.62~24.32	**1,3,4**
**Q.TGW-2B**	**AX-95244396**	TGW	E3	chr2B	7.49E+08	4.82~5	8.44~22.25	**1,4**
**Q.TGW-2D**	**AX-94779177**	TGW	E6	chr2D	14897557	3.91~4.25	5.16~6.42	**2,4,5**
**Q.TGW-3A**	**AX-94417800**	TGW	E5	chr3A	6.44E+08	5.05~7.09	6.18~9.64	**1,2,4,5**
**Q.TGW-3B**	**AX-94672617**	TGW	E6	chr3B	22056810	3.04~3.24	5.23~5.25	**2,4,5**
**Q.TGW-3B**	**AX-94761699**	TGW	E3	chr3B	1.10E+08	3.16~4.58	1.89~5.07	**2,4,5**
**Q.TGW-4D**	**AX-94988276**	TGW	E6	chr4D	1.02E+08	3.47~4.8	17.09~26.63	**2,4,5**
**Q.TGW-5A**	**AX-94803239**	TGW	E2	chr5A	6.39E+08	5.72~9.79	12.85~14.9	**1,2,**
**Q.TGW-5D**	**AX-95234313**	TGW	E4	chr5D	3.73E+08	3.8~3.92	10.59~21.45	**1,2,**
**Q.TGW-5D**	**AX-95250945**	TGW	E1	chr5D	4.80E+08	3.01~3.99	3.38~4.86	**1,2,**
**Q.TGW-6A**	**AX-95198425**	TGW	E2	chr6A	60739038	4.25~9.41	10.69~22.01	**2,4,**
**Q.TGW-6A**	**AX-94940257**	TGW	E1	chr6A	6.17E+08	4.47~5.91	5~10.39	**1,2,4**
**Q.TGW-7B**	**AX-94963644**	TGW	E4	chr7B	1256220	3.59~5.48	2.64~3.72	**2,4,**
**Q.TGW-7B**	**AX-94409804**	TGW	E3	chr7B	6.74E+08	3.32~4.6	2.39~7.4	**1,2,4**
**Q.TGW-7B**	**AX-94429451**	TGW	E5	chr7B	6.85E+08	4.1~5.59	11.96~23.87	**1,2,4,5**
**Q.TGW-7B**	**AX-95133915**	TGW	E4	chr7B	7.07E+08	3.14~4.26	1.81~6.63	**1,2,5**
**Q.TGW-7D**	**AX-94688812**	TGW	E1	chr7D	1.00E+08	3.77~6.35	2.99~5.14	**1,2,4**
**Q.GL-GLWR-6B**	**AX-94951003**	GL,GLWR	E1	chr6B	7.15E+08	3.48~4.01	0.03~2.72	**4**
**Q.GW-GLWR-2D**	**AX-94623084**	GW,GLWR	E7	chr2D	6.12E+08	3.88~6.55	8.09~28.53	**2,4,5,1**
**Q.GW-TGW-4A**	**AX-94395005**	GW,TGW	E5	chr4A	4.93E+08	4.68~7.58	0.65~20.96	**2,5,1,4,5**
